# SEEI: spherical evolution with feedback mechanism for identifying epistatic interactions

**DOI:** 10.1186/s12864-024-10373-4

**Published:** 2024-05-13

**Authors:** De-yu Tang, Yi-jun Mao, Jie Zhao, Jin Yang, Shi-yin Li, Fu-xiang Ren, Junxi Zheng

**Affiliations:** 1 Department of Computer Science, School of Mathematics and Informatics, School of Software Engineering, South China Agricultural University, Guangzhou, 510642 PR China; 2 School of Management, Guangdong University of Technology, Guangzhou, 510006 PR China; 3 School of Medical Information and Engineering, Guangdong Pharmaceutical University, Guangzhou, 510006 PR China

**Keywords:** Epistatic interactions, SNP, Spherical evolutionary, GWAS, Population updating strategy

## Abstract

**Background:**

Detecting epistatic interactions (EIs) involves the exploration of associations among single nucleotide polymorphisms (SNPs) and complex diseases, which is an important task in genome-wide association studies. The EI detection problem is dependent on epistasis models and corresponding optimization methods. Although various models and methods have been proposed to detect EIs, identifying EIs efficiently and accurately is still a challenge.

**Results:**

Here, we propose a linear mixed statistical epistasis model (LMSE) and a spherical evolution approach with a feedback mechanism (named SEEI). The LMSE model expands the existing single epistasis models such as LR-Score, K2-Score, Mutual information, and Gini index. The SEEI includes an adaptive spherical search strategy and population updating strategy, which ensures that the algorithm is not easily trapped in local optima. We analyzed the performances of 8 random disease models, 12 disease models with marginal effects, 30 disease models without marginal effects, and 10 high-order disease models. The 60 simulated disease models and a real breast cancer dataset were used to evaluate eight algorithms (SEEI, EACO, EpiACO, FDHEIW, MP-HS-DHSI, NHSA-DHSC, SNPHarvester, CSE). Three evaluation criteria (pow1, pow2, pow3), a T-test, and a Friedman test were used to compare the performances of these algorithms. The results show that the SEEI algorithm (order 1, averages ranks = 13.125) outperformed the other algorithms in detecting EIs.

**Conclusions:**

Here, we propose an LMSE model and an evolutionary computing method (SEEI) to solve the optimization problem of the LMSE model. The proposed method performed better than the other seven algorithms tested in its ability to identify EIs in genome-wide association datasets. We identified new SNP–SNP combinations in the real breast cancer dataset and verified the results. Our findings provide new insights for the diagnosis and treatment of breast cancer.

**Availability and implementation: **https://github.com/scutdy/SSO/blob/master/SEEI.zip.

**Supplementary Information:**

The online version contains supplementary material available at 10.1186/s12864-024-10373-4.

## Background

Complex disorders are defined as diseases that are driven by more than one genetic factor, including multiple genes, or gene–gene or gene–environment interactions [1]. High-throughput genotyping technologies have led to the rapid development of genome-wide association studies (GWAS), and high-risk loci of many complex disorders have been identified [2]. However, the reported loci explain only part of the genetic mechanism, and the interactions of single nucleotide polymorphisms (SNPs) or epistasis are considered to be important reasons for the “missing” heritability [3]. Epistatic interactions (EIs) are now considered to be one of the important genetic bases for the occurrence and development of complex diseases [4]. However, studying EIs is challenging because of the large number of SNPs that need to be tested and the huge amount of computation that is required [2]. Therefore, an effective and efficient epistasis detection method for 2-locus or multi-locus SNPs in the whole genome will be of great significance.

Many EI detection methods have been proposed. These methods can be classified as exhaustive search, stochastic search, machine learning, and meta-heuristic methods. The exhaustive search method systematically lists all possible candidates and explores all possible SNP combinations [5]. Multifactor dimensionality reduction (MDR) [6] is an exhaustive search method that simplifies high-dimensional genotype combination data into a single dimension. MDR is a model-free method that can identify gene–gene interactions in high-order data [7–9]. Extensions of MDR, such as MDR-ER [10], Cox-MDR [11], and MOMDR [12], have been proposed. Stochastic search methods such as random search and filtering methods also perform well. Random search methods include Bayesian epistatic association mapping (BEAM) [13]. Filtering methods reduce the computational burden by discarding large numbers of SNPs, and include EpiMiner [14], weighted heuristic anytime search [15], FDHEIW [16], LRMW [17], BADTrees [18], SNPRuler [19], ensemble learning-based approach (ELSSI) [20], EpiReSIM [21], MDSN [22], EpiMC [23], and modeling epistatic interaction (MEI) [24]. These methods sometimes screen out potentially meaningful single SNPs or SNP–SNP interactions, which reduces their detection power. Machine learning methods include random forest, support vector machine, and neural network [25, 26]. Methods based on machine learning generally have the problem that the results are difficult to interpret [27]. Meta-heuristic methods (swarm intelligence algorithms) based on Darwin’s theory of natural selection aim to imitate the behavior of various living beings in nature to find the optimal solution of complex problems. Meta-heuristic algorithms have become increasingly popular in solving EI detection problems. They include the cuckoo search epistasis (CSE) algorithm [28], MACOED [29], epiACO [30], MP-HS-DHSI [31], the extended ant colony optimization (EACO) algorithm [32], NHSA-DHSC [33], EIMOABC/D [34], Intelligent Privacy-Preserving (IPP) scheme [35], GEP-EpiSeeker [36], SFMOABC [37], EpiMOGA [38], and multi-objective evolutionary computation (MEC) [39].

Many meta-heuristic methods aim to solve a specific optimization epistasis model (single-objective model or multi-objective model), and therefore cannot be applied widely to different optimization epistasis models. For example, the CSE algorithm uses only the K2 score model, the epiACO algorithm uses only a mixed model of K2 score and mutual information (MI), the EACO algorithm combines only MI and the Gini index (GINI), the EIMOABC/D algorithm uses only the K2 score and GINI as a multi-objective model, and the GEP-EpiSeeker algorithm uses only the K2 score model. These optimization epistasis models have their own advantages and disadvantages when they are applied to EI problems on different datasets. Therefore, a meta-heuristic method that can be widely applied in multiple optimization epistasis models is needed to increase the generalization ability of the algorithms. When using meta-heuristic methods, the balance between exploitation and exploration is key to ensure that the algorithm escapes from the local optima and ultimately finds the global optimal solution. Furthermore, most meta-heuristic algorithms search in hypercube space (e.g., differential evolution, particle swarm optimization, artificial bee colony), which limits their search ability. In 2019, we proposed a spherical evolution algorithm that uses a super spherical search style that has a larger search space than the hypercube search style [40]. However, the standard spherical search algorithm can easily fall into a local optimum because it lacks a systematic feedback mechanism. To address these issues, we implemented the following innovations to solve the EI detection problem.

1. Develop a spherical evolution framework that uses the spherical search approach based on a feedback mechanism. Circle and spherical searches are executed alternately to solve any k-order (k ≥ 2) SNP combination problem. The individual arrangement and parameter adjustment of the search are dependent on a feedback mechanism.
2. Develop the feedback mechanism by spherical search scale parameter adjustment and a population updating strategy. The spherical search scale parameter is updated according to the winning ability of each individual for each generation. The excellent individuals in each generation are saved in a set and the population size can be decreased by linear reduction.
3. Apply a linear mixed optimization epistasis (LMOE) model as the fitness function. The LMOE model simplifies a multi-objective problem as a single-objective problem.

We tested the proposed spherical evolution approach with a feedback mechanism algorithm that we called SEEI on the single optimization epistasis model (single-objective optimization problem) and the LMOE model (multi-objective optimization problem) and verified its performance and generalization ability.

**Fig. 1:**
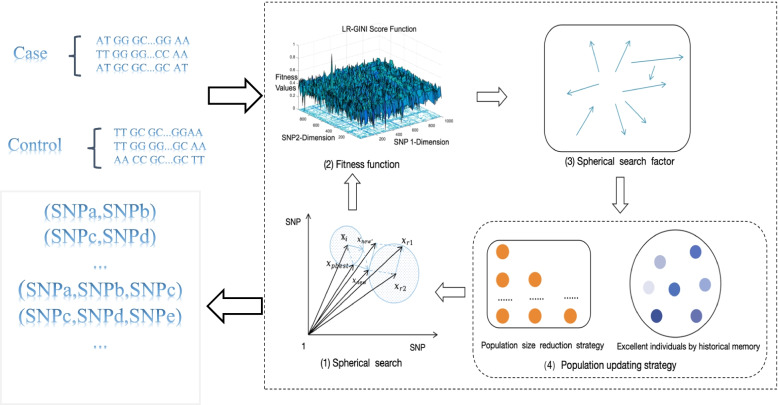
Framework of the SEEI algorithm. (1) Spherical search: spherical search operator in each generation. (2) Fitness function: LMOE model is used for LR and GINI. (3) Spherical search factor: arrows with different directions and lengths indicate the different search scales in each generation. (4) Population updating strategy: number of yellow balls in the rectangle area decrease gradually, indicating that the population size gradually decreased by a linear function. Different colored balls in the circle area indicate excellent individuals in each generation. The population updating strategy enhances the diversity of the population

The SEEI algorithm (Fig. 1) has four main components: spherical search, fitness function, spherical search factor, and population updating strategy. The spherical search components (Xi, Xbest, Xr1, Xr2) are used for two spherical searches. The fitness function uses the LMOE model, which can be used for single-objective and two-objective optimization models because it converts two-objective optimization problems to single-objective optimization problems. The spherical search factor can be considered as an adaptive parameter adjustment strategy in which the search factor parameter controls the spherical search scale; large scale values will make the search for large search space, whereas small scale values will make the search for small search space. Therefore, adaptive adjustment of the search scale is a key task of the SEEI algorithm.

## Experimental results and analysis

We tested the performance of the SEEI approach on 60 simulated datasets in various types of disease models and a real breast cancer dataset, and compared its performance with those of seven state-of-the-art algorithms, namely CSE [28], EACO [32], EpiACO [30], FDHEIW [16], MP-HS-DHSI [31], NHSA-DHSC [33], and SNPHarvester [41], on the same datasets. To ensure the fairness of the comparisons, we used the parameter settings of the algorithms from the original articles. Details of each algorithm are provided in Supplementary Files. To fully evaluate the performance of these algorithms in different disease models, three evaluation criteria were used, namely $Power1$, $Power2$, and $Power3$. $Power1$ is defined as $Power1=\frac{\#S}{\#T}$, where *#T* is the number of datasets generated by the same model (*#T* = 100 in our study), *#S* is the number of pathogenic datasets found from *#T* datasets. (The best SNP combination can be successfully detected in the dataset.) $Power2$ uses the G-test statistical method [33, 42] to test the significance level of candidate SNP combinations under $Power1$. G-test is a likelihood ratio test asymptotically similar to Pearson’s chi-square and superior to the approximation to the theoretical chi-square distribution. $Power3$ uses the balanced accuracy [7] and predictive error rate [43] of the MDR classifier under $Power1$. The three evaluation criteria were used to evaluate the ability of SEEI and the seven selected optimization algorithms to detect EIs.

### Parameter setting for the eight algorithms tested

For a fair evaluation of the comparisons, we used the running number of the fitness function with MAX_FES set as the maximum running number of the fitness function. The parameters of the eight algorithms were from the original papers. MAX_FES was set as: 2-order MAX_FES = 20000, 3-order MAX_FES = 20000 × 3, and 4-order MAX_FES = 20000 × 4. The parameter setting of each algorithm are as follows:

1. EACO: AntNumber = 200, evaporation = 0.3, Tau0 = 1, Alfa = 1, Beta = 1. AntNumber is the number of ants in the population, the initial pheromone Tau0 was set to 1, and Alfa and Beta, which determine the weights of pheromone and heuristic information, were set to 1. The evaporation coefficient of pheromones was set to 0.3.
2. MP-HS-DHSI: HMS = 10 for each HM, HMCR = 0.98, PAR = 0.35, threshold *p*-value for G-test = 1/Cnk, pre_error_rate = 0.45. Harmony memory (HM) is composed of harmonies. Harmony memory size (HMS) is the population size, harmony memory considering rate (HMCR) is the crossover rate, pitch adjusting rate (PAR) is the selection rate for different dimensions in each individual, and pre_error_rate is the MDR parameter.
3. NHSA-DHSC: HMS = 50, HMCR = 0.95, PAR = 0.35. Harmony memory (HM) is composed of harmonies. HMS is the population size, HMCR is the crossover rate, and PAR is the selection rate for different dimensions in each individual.
4. epiACO: AntNumber = 200; τ0 = 1, η = 1, α = 1, β = 1, evaporation coefficient ρ = 0.2, constant ξ = 0.3. AntNumber is the number of the ants in the population, τ0 is the pheromones of the path from position i to position j at iteration t. The heuristic information of the path from position i to position j is denoted as η. α and β are parameters that control the importances of pheromones and heuristic information respectively. The evaporation coefficient of pheromones was set to 0.2. Constant ξ is set as 0.3.
5. FDHEIW: K = epi_num, Candatiesize = 2 × epi_num. K is the number of SNP combination and Candatiesize is the number of candidate solution sets.
6. SNPHarvester: SuccessiveRun = 50. SuccessiveRun is the number of searches in each iteration.
7. CSE: Fraction of eggs discarded each generation = 0.25, maximum number of steps to take in a levy flight = 1, number of groups = 5, and number of nests = 100 (population size).
8. SEEI: pop_size = 50, p_best_rate = 0.11, arc_rate = 2.6, memory_size = 6, min_pop_size = 4. pop_size is the number of the population, p_best_rate is the rate of the best solutions in the population, arc_rate is the number of a population of outstanding individuals in history, memory_size is the search factor number in memory, and min_pop_size is the population size at the end of the final iteration.

### Experiments on simulated datasets

To comprehensively compare the eight algorithms (SEEI, EACO, EpiACO, FDHEIW, MP-HS-DHSI, NHSA-DHSC, SNPHarvester, CSE), we built 12 disease models with marginal effect, 30 disease models without marginal effect, and 8 random disease models. Details of the disease models are provided in Supplementary files.

#### Disease models with marginal effect (DMEs)

**Fig. 2:**
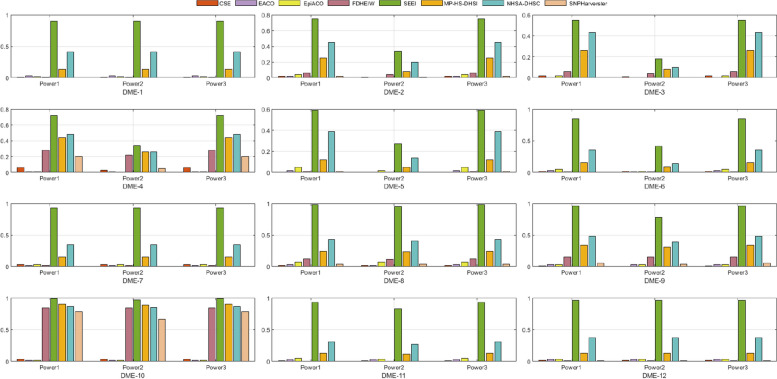
Performances of SEEI and seven other algorithms in 12 2-order disease models with marginal effect (DMEs). *Power*1, *Power*2, and *Power*3 were used to evaluate the performances. DME-1–DME-6, number of samples 800; DME-7–DME-12, number of samples 1600

Twelve DMEs were used to evaluate the performances of SEEI and the seven other algorithms for detecting EIs [9, 44, 45]. The DMEs were designed according to the interaction structure with different diseases. Details of the multi-locus penetrances are presented in Table S1. The heritability (h2) values were between 0.031 and 0.008. In each disease model, 100 datasets were randomly generated using GAMETES software, which can generate datasets containing a specific two-locus EI with random architectures. Each dataset included an interacting SNP pair (M0P0–M1P1) that was generated according to the disease model setting, and other SNPs are generated with minimum allele frequency (MAF) selected uniformly in (0.05, 0.5). For each DME, we simulated 100 replicate datasets with sample sizes 800 and balanced cases and controls (DME-1–DME-6), and sample sizes of 1600 and balanced cases and controls (DME-7–DME-12), and a total of 1000 SNPs. *Power*1, *Power*2, and *Power*3 values for the eight algorithms in the 12 DMEs were shown in Fig. 2. SEEI had higher *Power*1, *Power*2, and *Power*3 than the other seven algorithms in the 12 DMEs, indicating that the search ability of SEEI was better than that of the other seven algorithms. We also verified the robustness of the SEEI algorithm and found that it outperformed the other seven algorithms when the number of samples was increased from 800 (DME-1–DME-6) to 1600 (DME-7–DME-12). Indeed, the performances of all the other algorithms, except for that in DME-10, decreased when the number of samples was 1600. NHSA-DHSC and MP-HS-DHSI outperformed all the other algorithms, except SEEI.

#### Disease models without marginal effect (DNMEs)

**Fig. 3:**
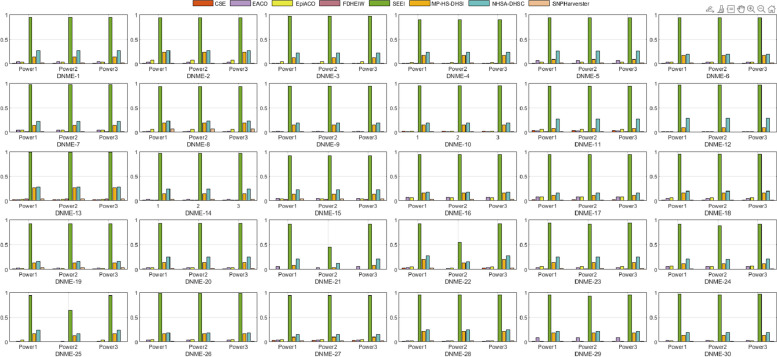
Performances of SEEI and seven other algorithms in 30 2-order disease models without marginal effects (DNMEs). *Power*1, *Power*2, and *Power*3 were used to evaluate the performances. DNME-1–DNME-30, number of samples 1600

Thirty 2-locus and pure disease models without marginal effects (DNMEs) were from a previous study [19]. The simulated datasets were generated with various parameter settings (heritability (h2) and MAF) using GAMETES [44]. Each dataset contained a specific 2-locus interacting SNP pair (M0P0–M1P1) with random architectures. The details of the multi-locus penetrances are presented in Table S2. The h2 values controlled the phenotypic variation of all disease models and ranged from 0.025 to 0.2, and the MAFs were 0.2 and 0.4. Each disease model was generated using 100 datasets consisting of 1000 SNPs, two of which (M0P0 and M1P1) were the specific SNPs. The other SNPs were generated with MAFs selected uniformly in (0.05, 0.5). For each selected DNME, we simulated 100 replicate datasets with sample sizes of 800 and balanced cases and controls, and a total of 1000 SNPs. We used 30 different DNMEs to fully test the detection ability of the eight algorithms. The performances of SEEI and the other seven algorithms in the 2-order DNMEs (DNME-1–DNME-30) are shown in Fig. 3. The SEEI algorithm outperformed the other seven algorithms, achieving the highest power among the DNMEs. These results again demonstrate the effectiveness of the SEEI algorithm in screening for significant SNP combinations.

#### Random disease models

**Fig. 4:**
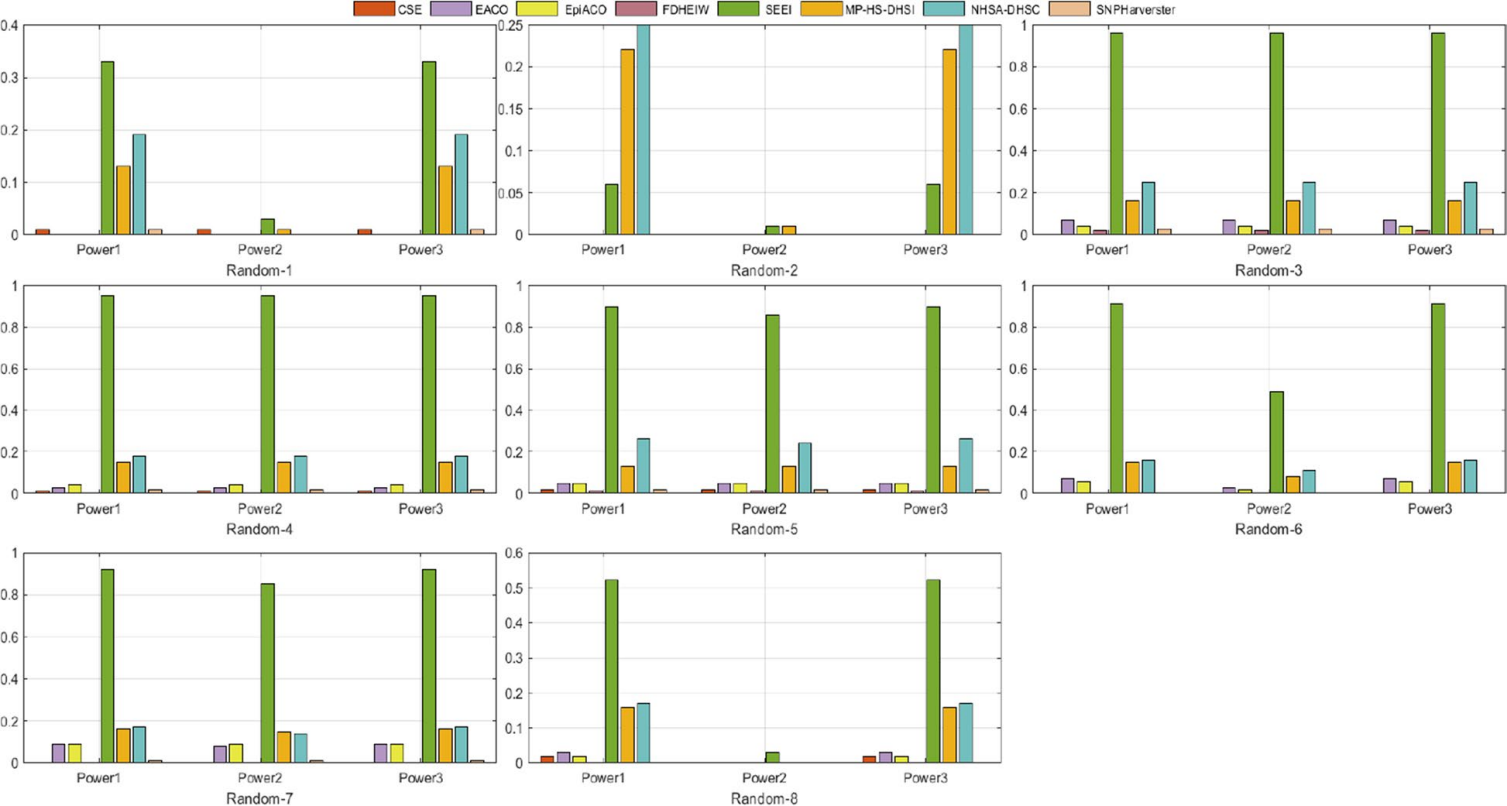
Performances of SEEI and seven other algorithms in eight 2-order disease models (Randoms). *Power*1, *Power*2, and *Power*3 were used to evaluate the performances. Random-1–Random-8, number of samples 1600

We used GAMETES to generate 100,000 random, strict, and pure disease models for each of the different combinations of genetic constraints that were obtained using different two-locus interacting SNP pairs (M0P0–M1P1); h2 values of 0.001, 0.025, 0.05, and 0.1, and MAFs of 0.2 and 0.4, with a varying population prevalence. For each setting, 100,000 disease models were ranked according to the ease of detection measure (EDM), and the eight disease models with the lowest EDM values were selected as the random disease models for data simulation (Random-1–Random-8). For each selected disease model, we simulated 100 replicate datasets with sample sizes 800 with balanced cases and controls and a total of 1000 SNPs. Each dataset contained one pair of highly interactive SNPs (M0P0–M1P1), and the other SNPs were generated with MAFs selected uniformly in (0.05, 0.5). *Power*1, *Power*2, and *Power*3 were higher for SEEI than they were for the other seven algorithms (Fig. 4), except in Random-2, where *Power*1 and *Powe*r3 were smaller for SEEI than they were for MP-HS-DHSI and NHSA-DHSC. In Random-1, the power of the eight algorithms was < 0.4, indicating that many algorithms were unable to detect the best SNP combination. For SEEI and NHSA-DHSC, *Power*1 was > 0.3 and < 0.2, respectively, implying that SEEI significantly outperformed NHSA-DHSC. In Random-2, only SEEI, MP-HS-DHSI, and NHSA-DHSC detected the best SNP combination.

#### High-order disease models (HODMs)

**Fig. 5:**
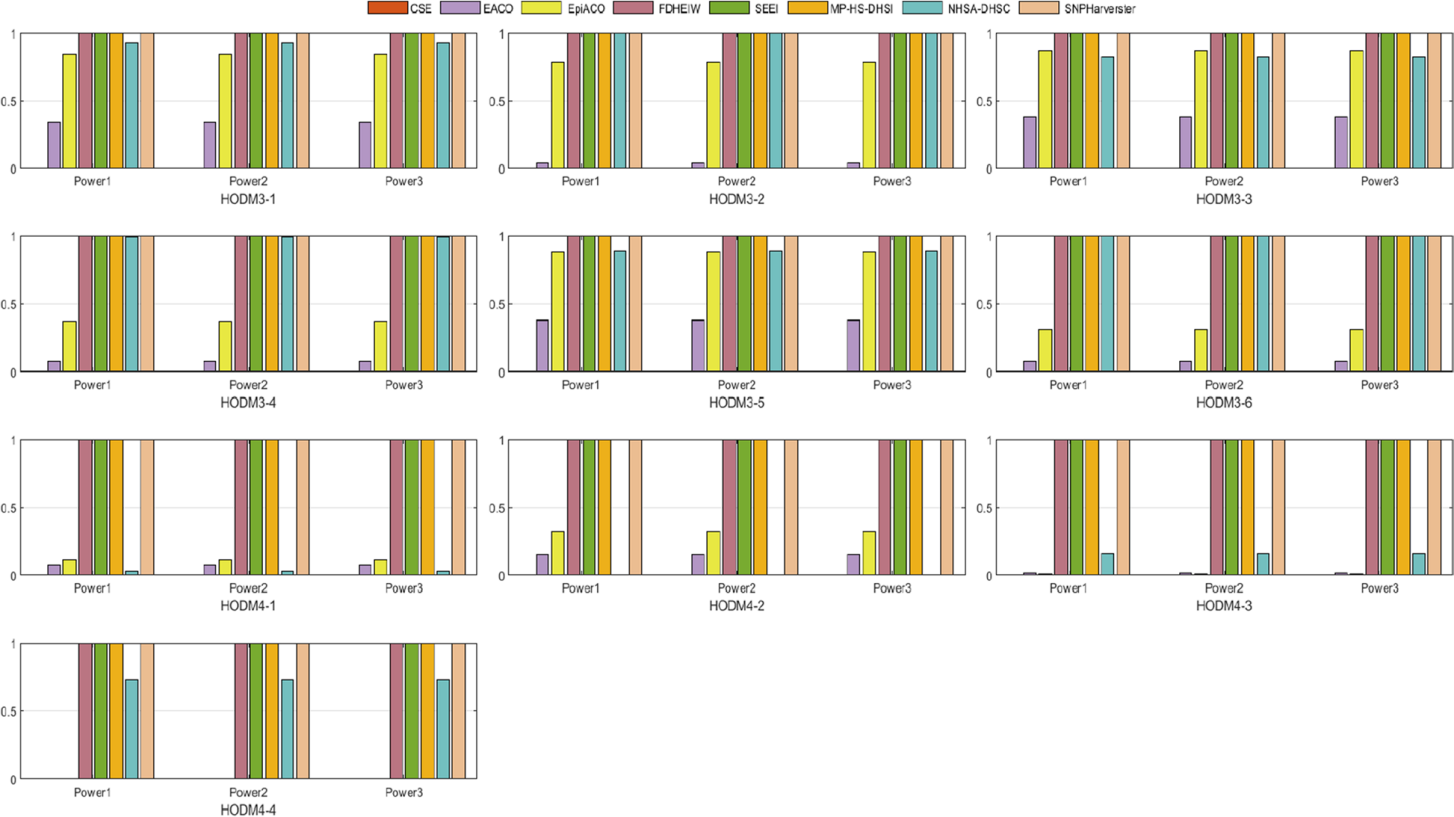
Performances of SEEI and seven other algorithms in 10 high-order disease models (HODMs). *Power*1, *Power*2, and *Power*3 were used to evaluate the performances. HODM3-1–HODM3-6, 3-order disease models; HODM4-1–HODM4-4, 4-order disease models. HODM3-1–HODM4-4, number of samples 500

We selected the additive models in literature [46] that define EIs with marginal effects. All the simulation configurations and datasets are publicly available at Github. (https://github.com/UDC-GAC/epistasis-simulation-data). Details of the multi-locus penetrances are presented in Table S3. The criteria to create penetrance tables of third and fourth-order were MAF values of 0.10, 0.25, and 0.40, and h2 values of 0.10, 0.25, 0.50, and 0.80 with prevalence > 1E − 06. From each penetrance table, 100 datasets were generated containing 500 SNPs from 2000 individuals (1000 cases and 1000 controls). We generated 10 simulated datasets; six for 3-order models and four for 4-order models. The performances of SEEI and the other seven algorithms on the 3-locus and 4-locus models are shown in Fig. 5. Most of the methods produced the highest power value (i.e., 1.0) on the high-order data models, confirming that these HODMs easily detected SNP–SNP interactions. SEEI, FDHEIW, MP-HS-DHS, and SNPHarvester produced the best performances, whereas CSE performed the worst. These results show that among the 3-locus and 4-locus models, SEEI was highly effective in detecting high-order interactions.

#### Comparison of the different disease models

To comprehensively validate the performance of our proposed method, the four disease models, DMEs, DNMEs, Random models, and HODMs were used to compare SEEI and the seven state-of-the-art algorithms, EACO, MP-HS-DHSI, NHSA-DHSC, epiACO, FDHEIW, SNPHarvester, and CSE. In the 12 DME models (Fig. 2), SEEI, NHSA-DHSC, MP-HS-DHSI, and FDHEIW had better *pow1*, *pow2*, and *pow3* values than the other algorithms. In addition, the power values of SEEI increased when the number of samples was increased from 800 (DME-1–DME-6) to 1600 (DME-7–DME-12). Conversely, the power values of some methods, such NHSA-DHSC, decreased when the number of samples was increased from 800 (DME-1–DME-6) to 1600 (DME-7–DME-12). In the 30 DNME models, SEEI, NHSA-DHSC, MP-HS-DHSI, epiACO, and EACO had the best pow1, pow2, and pow3 values (Fig. 3). The epiACO and EACO algorithms had better power values in the DNME models than they had in the DME models, whereas the FDHEIW had better power values in DME models than it had in DNME models. In the eight random disease models, CSE, FDHEIW, and SNPHarvester had very low power values and failed to detect the epistatic interaction pairs, whereas SEEI, NHSA-DHSC, MP-HS-DHSI, epiACO, and EACO had better power values (Fig. 4). Only the SEEI, NHSA-DHSC, and MP-HS-DHSI algorithms had high stable power values in the 2-order DME, DNME, and random disease models. In the 10 high-order disease models (HODMs), only SEEI, FDHEIW, MP-HS-DHS, and SNPHarvester had high power values (Fig. 5). The detection ability of some methods, such as NHSA-DHSC, decreased from the 2-order models to the high order models, whereas the detection ability of FDHEIW, MP-HS-DHS, and SNPHarvester increased from the 2-order models to the high order models. This result indicates that FDHEIW, MP-HS-DHS, SNPHarvester, and NHSA-DHSC are more suitable for certain disease models. SEEI performed better than all the other seven methods in the 2-order and a high order models, demonstrating that SEEI has strong robustness and can be used for many different types of disease models.

#### Comprehensive analysis of the 60 disease models

**Fig. 6:**
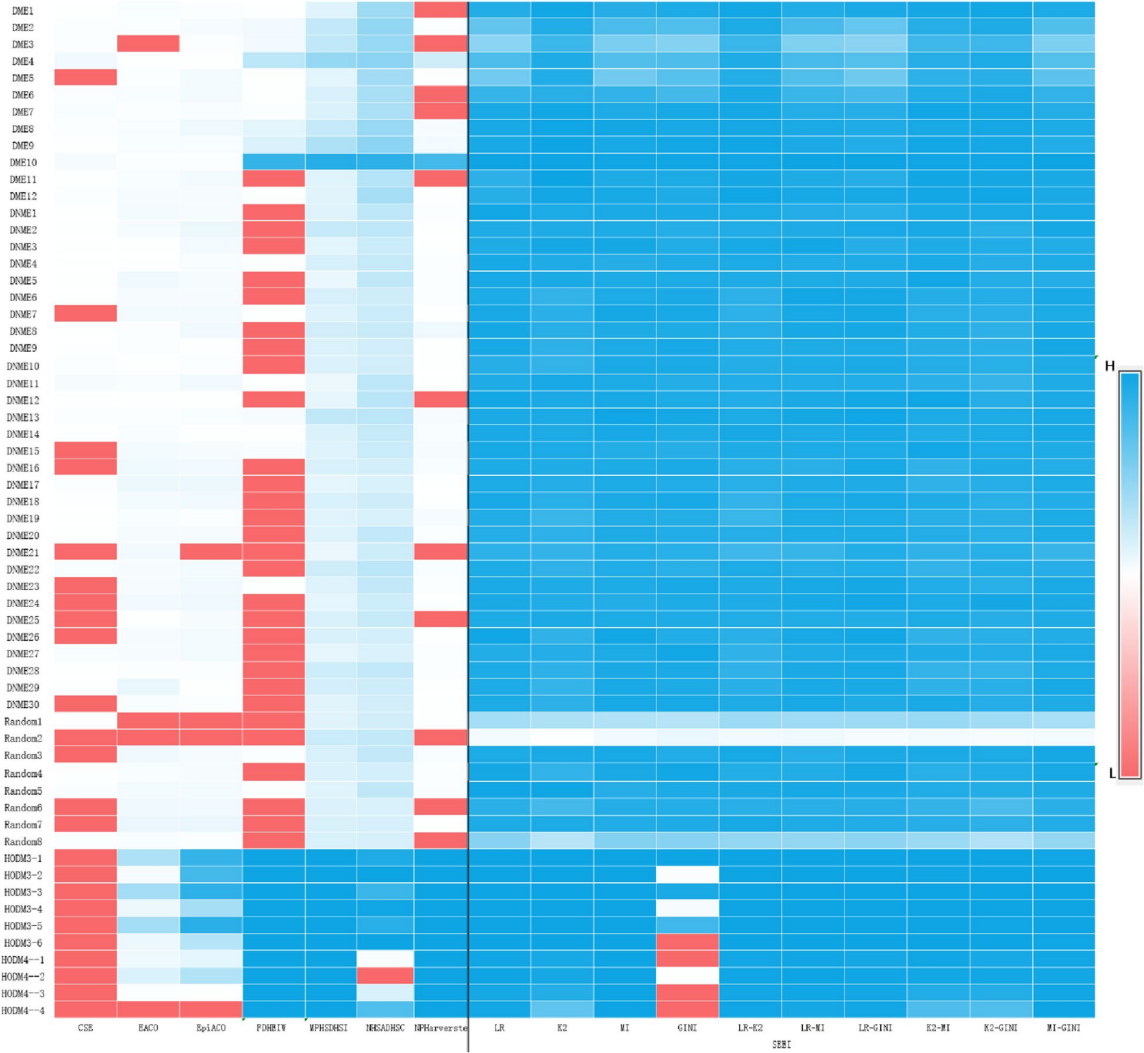
Heap map of 17 algorithms in the 60 disease models. The algorithms include the seven algorithms tested (CSE, EACO, EpiACO, FDHEIW, MP-HS-DHS, NHSA-DHSC, SNPHarvester) and the SEEI algorithm with 10 epistasis optimization methods. The disease models were the DMEs, DNMEs, Random, and HODMs. The 10 SEEI methods included four single epistasis optimization methods (LR, K2, MI, GINI) and six combination epistasis optimization methods (LR-K2, LR-MI, LR-GINI, K2-MI, K2-GINI, MI-GINI). *Power*1 was used to evaluate the performances. Red, white, and blue blocks indicate low, moderate, and high power, respectively

The heap map (Fig. 6) shows the detection powers of the seven algorithms and the SEEI algorithm with 10 epistasis optimization methods in the 60 disease models. SEEI with 10 epistasis optimization methods outperformed the other seven algorithms in the 50 2-order disease models, with the exception of MP-HS-DHS and NHSA-DHSC in the Random-2 model, which outperformed the other methods. In the 10 high-order disease models, SEEI (with 9 of the epistasis optimization methods), FDHEIW, MP-HS-DHS, and SNPHarvester outperformed the other algorithms. The results also show that the SEEI algorithm had the best performance in the 60 disease models with not only the single epistasis optimization models but also with the linear maxed epistasis optimization models.

The *Power*1 values for the eight algorithms, SEEI, EACO, EpiACO, FDHEIW, MP-HS-DHSI, NHSA-DHSC, SNPHarvester, and CSE, in the 60 disease models by T-test are shown in Table 1. The results show that SEEI outperformed the other seven algorithms. The average ranks of the 17 algorithms by Friedman test [47] are listed in Table 2. The detection power of the 10 SEEI epistasis optimization methods outperformed those of the other seven algorithms (*p*-value = 7.56E − 12; Table 3), showing that SEEI had better robustness and scalability than the other methods. This finding suggests that the SEEI algorithm may be applicable to other epistasis optimization models.

**Table 1:** *Power*1 values for eight algorithms in 60 disease models by T-test

	**EACO%**	**EpiACO%**	**FDHEIW%**	**MPHSDHSI%**	**NHSADHSC%**	**SNPHarverster%**	**CSE%**	**SEEI%**
**D01**	3	2	1	13	41	0	1	**90**
**D02**	2	4	6	25	45	2	2	**75**
**D03**	0	2	6	26	43	0	2	**55**
**D04**	1	1	28	44	48	20	6	**72**
**D05**	2	5	1	12	39	1	0	**59**
**D06**	3	5	1	16	36	0	2	**85**
**D07**	2	3	2	15	35	0	3	**93**
**D08**	3	7	12	24	43	4	2	**98**
**D09**	3	3	15	34	48	5	1	**96**
**D10**	2	2	84	90	87	78	4	**99**
**D11**	3	5	0	13	31	0	1	**93**
**D12**	4	4	1	13	37	1	2	**96**
**D13**	5	4	0	14	27	2	1	**95**
**D14**	4	8	0	24	27	1	1	**94**
**D15**	1	5	0	12	22	1	1	**97**
**D16**	1	3	1	17	24	2	1	**90**
**D17**	7	4	0	9	26	2	1	**94**
**D18**	4	4	0	17	20	2	1	**94**
**D19**	5	5	1	14	22	1	0	**97**
**D20**	2	6	0	19	23	7	1	**93**
**D21**	2	1	0	15	19	1	1	**94**
**D22**	1	2	0	15	19	1	2	**95**
**D23**	3	6	1	8	27	1	4	**94**
**D24**	1	1	0	10	29	0	1	**96**
**D25**	2	3	4	26	28	4	2	**99**
**D26**	3	1	1	15	24	3	1	**96**
**D27**	5	4	3	14	22	4	0	**91**
**D28**	7	6	0	16	18	3	0	**94**
**D29**	8	8	0	11	16	1	2	**94**
**D30**	5	6	0	16	20	1	1	**95**
**D31**	3	2	0	13	16	4	1	**92**
**D32**	4	4	0	14	25	2	1	**93**
**D33**	6	0	0	9	21	0	0	**91**
**D34**	4	5	0	20	28	3	3	**92**
**D35**	4	6	1	14	25	2	0	**94**
**D36**	6	7	0	11	21	1	0	**91**
**D37**	1	4	0	16	24	0	0	**94**
**D38**	4	5	0	16	18	1	0	**98**
**D39**	4	5	0	10	15	2	3	**94**
**D40**	2	2	0	21	25	2	1	**95**
**D41**	9	1	0	18	21	1	1	**95**
**D42**	3	2	0	13	19	1	0	**96**
**D43**	0	0	0	13	19	1	1	**33**
**D44**	0	0	0	22	**25**	0	0	6
**D45**	7	4	2	16	25	3	0	**96**
**D46**	3	4	0	15	18	2	1	**95**
**D47**	5	5	1	13	26	2	2	**90**
**D48**	7	6	0	15	16	0	0	**91**
**D49**	9	9	0	16	17	1	0	**92**
**D50**	3	2	0	16	17	0	2	**52**
**D51**	34	84	**100**	**100**	93	**100**	0	**100**
**D52**	4	78	**100**	**100**	**100**	**100**	0	**100**
**D53**	38	87	**100**	**100**	82	**100**	0	**100**
**D54**	8	37	**100**	**100**	99	**100**	0	**100**
**D55**	38	88	**100**	**100**	89	**100**	0	**100**
**D56**	8	31	**100**	**100**	**100**	**100**	0	**100**
**D57**	7	11	**100**	**100**	3	**100**	0	**100**
**D58**	15	32	**100**	**100**	0	**100**	0	**100**
**D59**	2	1	**100**	**100**	16	**100**	0	**100**
**D60**	0	0	**100**	**100**	73	**100**	0	**100**
	**3.04e-44**	**4.97e-34**	**2.30e-21**	**5.16e-20**	**7.59e-23**	**1.170e-21**	**6.42e-45**	**T-test** ***P*-values**

Rows D01–D60 contain *Power*1 percentages in the 60 disease models. Bold font indicates the best *Power*1 values among the eight algorithms. The last row shows the T-test *p*-values for SEEI measured against the other seven algorithms

**Table 2:** Average ranks by Friedman test analysis of the 17 algorithm in the 60 disease models

Order	Algorithm	Averages ranks
1	MI	13.125
2	LR	12.81
3	LR-GINI	12.75
4	LR-MI	12.53
5	MI-GINI	12.15
6	LR-K2	12.03
7	K2-MI	11.73
8	K2	11.56
9	GINI	11.04
10	K2-GINI	10.80
11	MP-HS-DHSI	7.35
12	NHSA-DHSC	6.92
13	SNPHarvester	4.50
14	EpiACO	3.99
15	FDHEIW	3.85
16	EACO	3.65
17	CSE	2.20

**Table 3:** Statistics of the Friedman test analysis for the 17 algorithms

Method	Statistical value	*p*-value
Friedman test	621.116	7.56E − 12

**Fig. 7:**
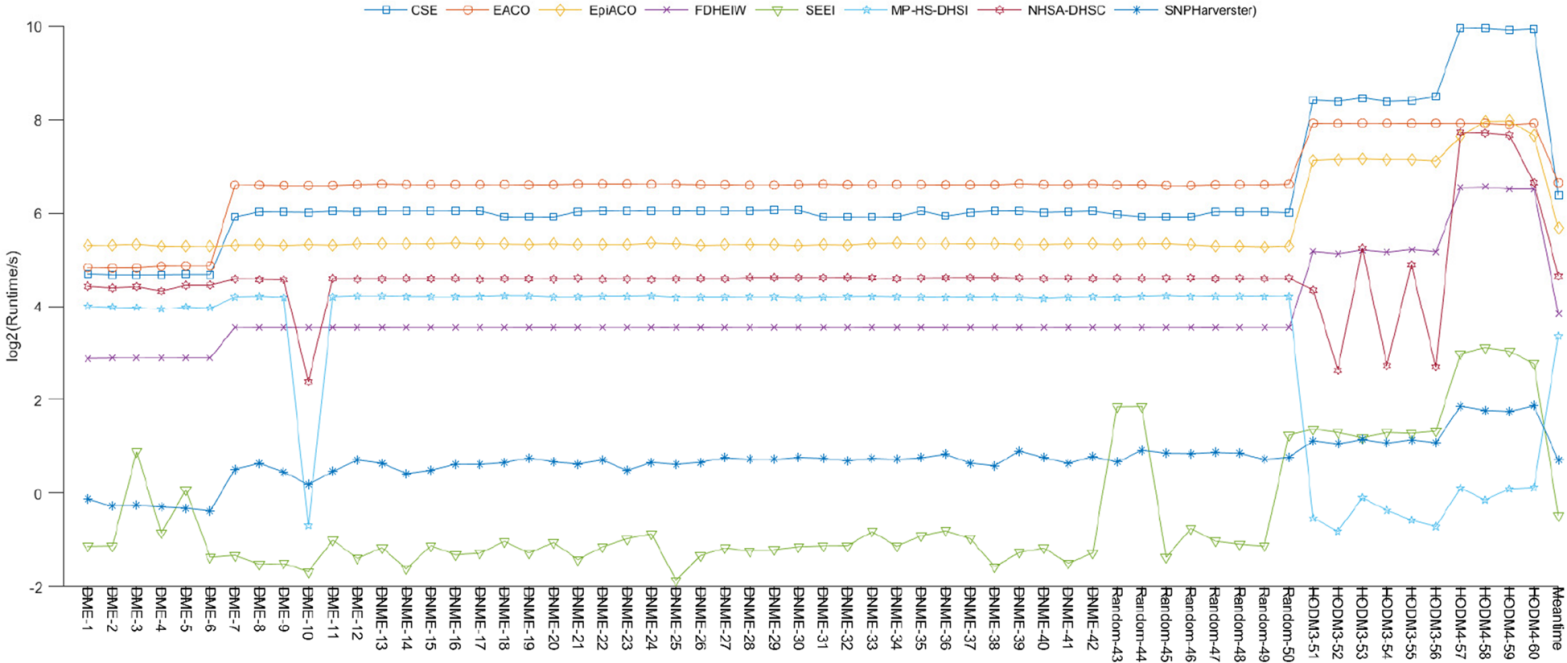
Running times and average running time for the eight algorithms in 60 disease models. The proposed SEEI algorithm effectively reduced the computational burden and had better detection ability than most similar algorithms

Because of the long running time required for high-order data, the gap in the running times between the high-order disease models for the other disease models was very large, and therefore we used the logarithm of the running times of all the algorithms for a better comparison. Compared with the other seven algorithms, the SEEI algorithm (MI) had the shortest running time in most of the disease models (Fig. 7). Although the running time of the SEEI (MI) algorithm was slightly longer than that of the SNPHarvester algorithm in DME-3, DME-5, Random-43, Random-44, and Random-50, and slightly longer than the MP-HS-DHSI and SNPHarvester algorithms in the high-order disease models, the average running time of SEEI was the shortest among the 60 disease models, implying that the SEEI algorithm was more adaptable to different disease models and had a lower computational burden than the other algorithms. We believe that the SEEI algorithm has promise in detecting disease-causing SNP combinations and has a broad future.

### Experiments on a real dataset

We used the breast cancer (BC) dataset of the Welcome Trust Case Control Consortium (WTCCC) project as the real dataset to evaluate the ability of SEEI to detect EIs [48]. Mutual information (MI) was used as the fitness function of SEEI. BC is a complex disease and its etiology is not fully understood [49]. The BC dataset included 15,436 SNPs from 1045 patients with BC and 1438 normal individuals from the 1958 birth cohort. Samples with SNP genotype deletion rates ≥ 2% were excluded, and for a SNP, if the genotype deletion rate of all the samples was ≥ 5%, or its *p*-value (Hardy–Weinberg equilibrium) was < 0.0001 in the controls, or the MAF was < 0.1, the SNP was excluded. After quality control, 3386 SNPs from 1045 patient and 1329 control samples in the BC dataset were used in this study.

**Fig. 8:**
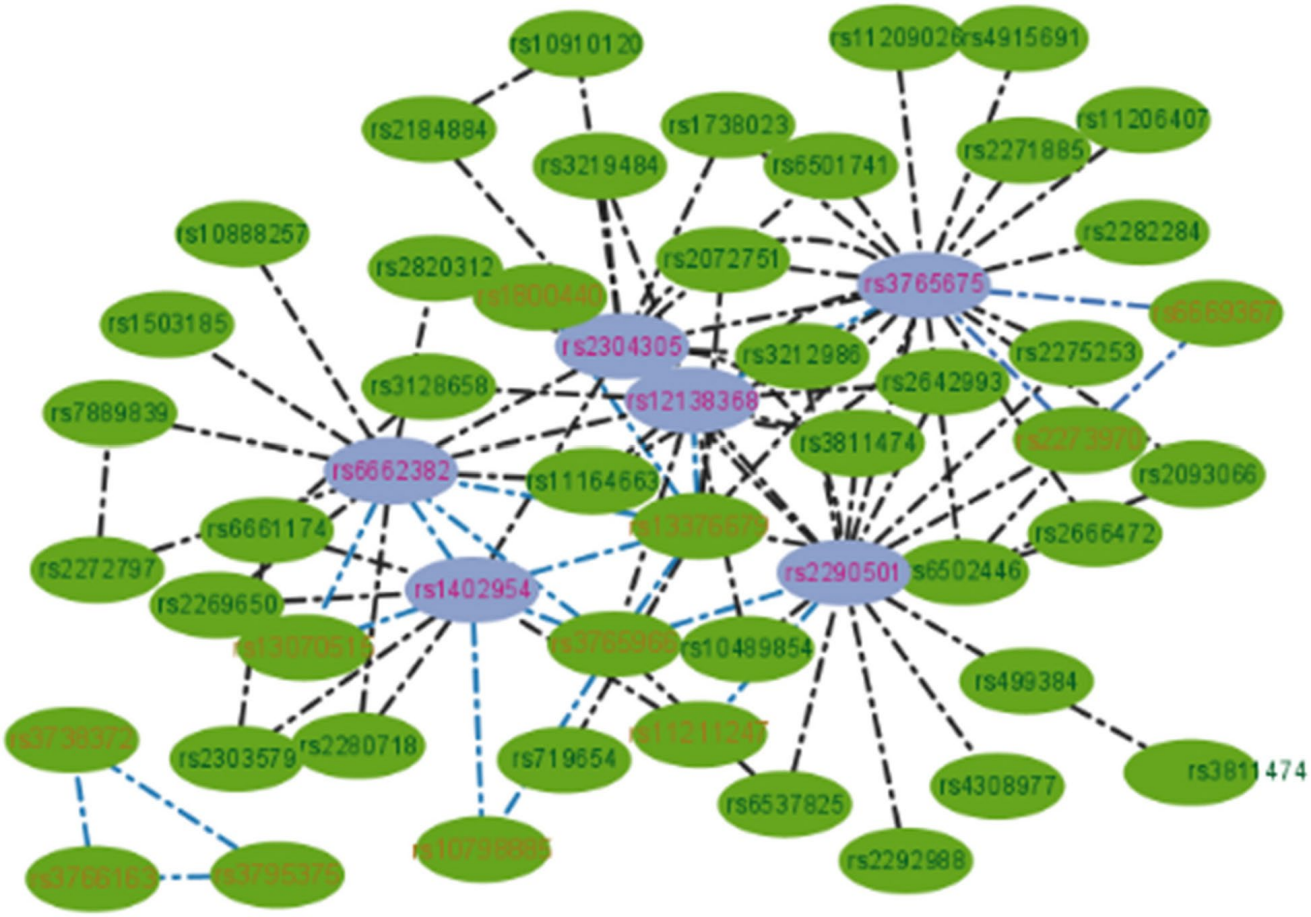
SNP interaction network for breast cancer. Nodes are SNPs and edges are SNP–SNP interactions. Black edges indicate two-way SNP interactions, and every two edges of the same color in the remaining edges connect three nodes, indicating three-order SNP interactions

The population size of SEEI was set to 400 and the maximum number of iterations was set to 5000. The SEEI algorithm identified some 2-way and 3-way SNP combinations that may be associated with BC (Table 4 and Fig. 8)*.* The single SNP (rs1402954) that was detected most frequently in the two-way SNP combinations is located in *FBXO3* on chromosome 11. FBXO3 is involved in ΔNp63α degradation to empower TGF-β signaling in promoting tumor metastasis and the TβRI–FBXO3–ΔNp63α axis is critically important in BC development and its clinical prognosis [50]. SNP rs2290501 is located in *HSPG2* on chromosome 1. HSPG2 is involved in tumor development and progression, and, in BC, HSPG2 was fragmented or completely lost in epithelial basement membrane, with a marked increase in HSPG2 abundance in the stroma [51]. SNP rs11211247 is located in *MAST2* on chromosome 1. Gene rearrangements in the microtubule-associated serine-threonine kinase (MAST) gene family have been identified in 5%–7% of invasive BCs [52]. SNP rs3765966, which is located in *CA6* on chromosome 1, was detected more than once in 3-way SNP combinations. *CA6* is highly expressed by many BCs [53]. SNP rs6662382 was also detected in 3-way SNP combinations. The relationship of rs6662382 with BC has not been reported so far, implying that this may be a new SNP combination associated with BC. The SNP interaction network for BC is shown in Fig. 8.

**Table 4:** Significant two-way and three-way SNP combinations identified in the WTCCC breast cancer dataset

SNP Combinations	Chromosome and Related Genes	Interaction G-test	*p*-Value
rs2290501;rs11211247	Chr1:HSPG2;Chr1:MAST2	0	< 1E-100
rs1402954;rs13376679	Chr11:FBXO3;Chr1:STIL	0	< 1E-100
rs12138368;rs3765966	Chr1:CATSPER4;Chr1:CA6	0	< 1E-100
rs2290501;rs3765675	Chr1:HSPG2;Chr1:TNNI3K	0	< 1E-100
rs2273970;rs6669367;rs3765675	Chr1:GALNT2;Chr1:DTL;Chr1:TNNI3K	0	< 1E-100
rs3765966;rs1402954,rs10798885	Chr1:CA6;Chr11:FBXO3;Chr1:COL16A1	0	< 1E-100
rs13376679;rs3765966;rs6662382	Chr1:STIL;Chr1:CA6;Chr1:OR10T2	0	< 1E-100
rs6662382;rs1402954;rs13070515	Chr1:OR10T2;Chr11:FBXO3;Chr3:LRRC15	0	< 1E-100
rs13376679;rs12138368;rs1800440	Chr1:STIL;Chr1:CATSPER4;Chr2:CYP1B1	0	< 1E-100
rs3766160;rs2791494;rs11939575	Chr1:CELA2B;Chr1:CLCA1;Chr1:FAT1	0	< 1E-100
rs3795375;rs3766163;rs3738372	Chr1:MAP3K21;Chr1:RSC1A1;Chr1:CAPN2	0	< 1E-100
rs13376679;rs3765675;rs2072751	Chr1:STIL;Chr1:TNNI3K;Chr1:VWA5B1	0	< 1E-100

## Discussion

We proposed a spherical evolution with feedback mechanism method called SEEI to effectively detect 2-order and high-order epistasis from genome-wide case–control data. To verify the robustness and scalability of SEEI, we used a linear mixed statistical epistasis model that converts the multi-objective problem to a single-objective problem. SEEI was compared with seven state-of-the-art algorithms (EACO, EpiACO, FDHEIW, MP-HS-DHSI, NHSA-DHSC, SNPHarvester) for four single epistasis models (LR, K2, MI, GINI) and six linear mixed statistical epistasis models (LR-K2, LR-MI, LR-GINI, K2-MI, K2-GINI, MI-GINI). We found that SEEI outperformed the other seven comparison methods for detection accuracy on simulated and real datasets. Furthermore, SEEI showed strong robustness and scalability, indicating that it can be used for both single and linear mixed statistical epistasis models. The proposed SEEI method performed well in identifying EIs, in particular, in solving multiple different epistasis optimization models, indicating that the SEEI algorithm has good generalization ability. However, the good performance of SEEI in all disease models or all real datasets cannot be guaranteed. We found that the SEEI algorithm still falls into local optima when tackling different kinds of data. To overcome these problems, further focus on the feedback mechanism of meta-heuristic methods, multi-subpopulation search methods, and the ensemble learning framework for different algorithms may help to greatly improve the generalization ability of algorithms in the future.

## Methods

### Related work

In the last four years, the EI identification problem has developed rapidly. The main focus has been on how to establish an epistasis interaction model and how to solve the model. Stochastic search methods and meta-heuristic methods are still hot topics in current research. Some models and search approaches for stochastic search methods have been proposed. Sun et al. [22] proposed a module detection method in which a SNP network was constructed and a node was comprehensively evaluated by the topological characteristics of the neighborhood. Wang et al. [23] proposed a matrix factorization based multiple clustering algorithm to generate multiple diverse clusters and then used Jaccard similarity to obtain candidate sets. David et al. [24] proposed a model that scores candidate SNP sets by computing maximum likelihood distribution for the observed phenotypes. Different from stochastic search method, meta-heuristic method is the general term for several optimization algorithms that are inspired by the Darwinian principles of nature’s capability to evolve organisms that are well adapted to their environment. Because of its strong search ability, many meta-heuristic methods have been used to solve the EI identification problem. Tuo et al. [31] proposed a multi-population harmony search (HS) algorithm and multiple criteria (Bayesian network-based K2-score, Jensen-Shannon (JS) divergence, likelihood ratio (LR) and normalized distance with joint entropy (ND-JE)) were adopted by four harmony memories to improve its ability to discriminate diverse disease models. Sun et al. [32] detected epistatic interactions based on the ant colony optimization (ACO) algorithm, the highlights of which were the introduced heuristic information, fitness function, and a candidate solutions filtration strategy. Two functionally complementary fitness functions, mutual information, and the Gini index, were combined to effectively evaluate the associations between SNP combinations and the phenotype. Li et al. [34] proposed and formulated an epistatic interaction multi-objective artificial bee colony algorithm based on decomposition (EIMOABC/D) to address those problems for genetic interaction detection in GWAS. First, to direct the genetic interaction detection, two objective functions were formulated to characterize various epistatic models, and a rank probability model was proposed to sort each population into different nondomination levels based on the fast nondominated sorting approach. Wang et al. [35] proposed a method based on the ACO algorithm to detect SNP–SNP interactions for GWAS, an IPP scheme. Initially, a multi-objective search algorithm was designed to discover candidate SNP sets related to a disease phenotype, which used a Differential Privacy method by disturbing the multi-objective function to construct a rational epistatic privacy protection strategy. Peng et al. [36] proposed several tailor-made chromosome rules to describe SNP combinations, and incorporated Bayesian network-based fitness evaluation into the evolution of tailor-made chromosomes to find candidate SNP combinations, and adopted the chi-square test to identify optimal solutions from suspected SNP combinations. Gu et al. [37] proposed a multi-objective artificial bee colony (ABC) algorithm based on the scale-free network (SFMOABC), which incorporated the scale-free network into the ABC algorithm to guide the update and selection of solutions. In addition, SFMOABC used the mutual information (MI) and the K2-Score of the Bayesian network as objective functions, and the opposition-based learning strategy was used to improve the search ability. Chen et al. [38] proposed a multi-objective genetic algorithm (EpiMOGA) for SNP epistasis detection. The K2 score based on the Bayesian network criterion and the Gini index of the diversity of the binary classification problem were used to guide the search process of the genetic algorithm. M. G-CJ et al. [39] proposed the application of two of the most successful multi-objective evolutionary algorithms to solve the binary classification problem, namely the reference-point based Many-objective Fast Non-dominated Sorting Genetic Algorithm (NSGA-III) and a Multi-objective Evolutionary Algorithm based on Decomposition with Dynamical Resource Allocation (MOEA/D-DRA). Yang et al. [12] proposed a novel ensemble learning-based approach (ELSSI) that can significantly reduce the bias of individual detectors and their computational load. ELSSI randomly divides SNPs into different subsets and evaluates them by multi-type detectors in parallel.

Clearly, a large number of many-objective meta-heuristic methods have been used to solve EI problems. The classical ABC algorithm, genetic algorithm, ant colony optimization, harmony search algorithm, and many epistasis interaction models (e.g., K2-score, JS, LR, MI) are among those that have been used. Novel meta-heuristic algorithms and epistasis interaction models have not yet been developed. We considered the spherical evolution (SE) method for solving the EI problem. The SE algorithm was first proposed by our team in 2019 [40]. Although the SE algorithm performed well in solving optimization problems, it was easily trapped in local optima. Here, we propose a SE method based on the feedback mechanism and a linear mixed statistical epistasis model (LMSE) instead of the traditional many-objective epistasis models that have been used to solve the EI problem.

### EI identification problem

The EI identification problem can be represented as a matrix D = (X,Y),$X\in {\mathbb{Z}}^{ m\times n}$, $Y\in {\mathbb{S}}^{ m\times 1}$, where *m* is the number of samples and *n* is the number of SNPs. In X ($\forall {x}_{i,j}\in \{\mathrm{0,1},2\}$, 0 represents the homozygous major allele, 1 represents the heterozygous allele, and 2 represents the homozygous minor allele, and ${x}_{i,j}$ is the genotype of the *j*-th SNP and the *i*-th sample in dataset D. In Y ($\forall {y}_{i}\in \{\mathrm{0,1}\}$), 0 represents the control and 1 represents the case, and ${y}_{i}$ is the phenotype of the *i*-th sample in dataset D, which represents the disease status of the *i*-th sample corresponding to its SNPs.

If the k-SNP combination set is defined as ${X}_{m,k}^{\mathrm{^{\prime}}}=\left\{({x}_{1..m,{j}_{1}},{x}_{1..m,{j}_{2}}...{x}_{1..m,{j}_{p}}...{x}_{1..m,{j}_{k}})|1\le p\le k,k<<n\right\}$, then the optimization epistasis model can be represented as: ${\min}_{x' \subset x}f\left(x'_{m,k},Y_m\right)$, where ${{\varvec{X}}\mathrm{^{\prime}}}_{{\varvec{m}},{\varvec{k}}}$ is a set of k-order SNPs and ${\varvec{f}}$ is an optimization epistasis model (a scoring function) for evaluating the association between a k-SNPs (${{\varvec{X}}\mathrm{^{\prime}}}_{{\varvec{m}},{\varvec{k}}}$) and disease status (${Y}_{m}$).

### Spherical evolution approach with feedback mechanism

The SE algorithm [40] searches for all regions of a sphere by continuously adjusting the radius and angle of the sphere. However, the current SE algorithm lacks a good feedback mechanism. Adaptive parameter control and population size control strategies have been proposed to improve the accuracy of the differential evolution algorithm [54]. Inspired by these strategies, we used adaptive spherical search and population updating strategies for the feedback mechanism of the SE algorithm. We also designed a fitness function using a linear mix optimization epistasis model. The main steps are:

- Step 1: Population initialization

Randomly generate N individuals (solution vectors) in the search space. In the ${{\varvec{j}}}_{{\varvec{t}}{\varvec{h}}}$ dimension, the search space is limited in [${a}_{\mathit{min},j}$,${a}_{\mathit{max},j}$], where ${a}_{\mathit{min},j}$ is 1 and ${a}_{\mathit{max},j}$ is equal to the order of SNP. If ${a}_{i,j}$ denotes a solution vector $i\in \left[\mathrm{1,2},\dots ,{\text{N}}\right],j\in [\mathrm{1,2}\dots ,\mathrm{ SNP}$], then

1 $${a}_{i,j}={a}_{\mathit{min},j}+rand[\mathrm{0,1}].({a}_{\mathit{max},j}-{a}_{\mathit{min},j})$$

where $rand[\mathrm{0,1}]$  is a number of uniform distribution from 0 to 1.

- Step 2: Spherical search operator

To obtain a better individual (solution vector), each individual ${a}_{i,*}^{g}$ in the population performs a spherical search operation as shown in Eqs. (5), (6), and (7) or Eqs. (8) and (9), where ${a}_{pbest,j}$ is an individual chosen from a set of the better individuals in the current generation (sort and select based on the better fitness values), ${a}_{r1,j}$ is an individual selected randomly from the population, and ${a}_{h,j}$ is an individual selected randomly from a set of the excellent individuals in each generation. $Scale$ is the only important parameter (search factor), which represents the radius of a sphere. In three-dimensional (3D) space, the spherical search can be formulated as:

2 $${a}_{i,j}^{g+1}={a}_{i,j}^{g}+Scale\cdot ||{a}_{pbest,*}-{a}_{i,*}|{|}_{2}\cdot {\prod }_{k=j}^{dim-1}{\text{sin}}\left({\theta }_{j}\right)+Scale\cdot ||{a}_{r1,*}-{a}_{h,*}|{|}_{2}\cdot {\prod }_{k=j}^{dim-1}{\text{sin}}\left({\theta }_{j}\right),j=1$$

3 $${a}_{i,j}^{g+1}={a}_{i,j}^{g}+Scale\cdot ||{a}_{pbest,*}-{a}_{i,*}|{|}_{2}\cdot {\prod }_{k=j}^{dim-1}{\text{sin}}\left({\theta }_{j}\right)+Scale\cdot ||{a}_{r1,*}-{a}_{h,*}|{|}_{2}\cdot {\prod }_{k=j}^{dim-1}{\text{sin}}\left({\theta }_{j}\right),j=1$$

4 $${a}_{i,l}^{g+1}={a}_{i,l}^{g}+Scale\cdot ||{a}_{pbest,*}-{a}_{i,*}|{|}_{2}.{\text{cos}}({\theta }_{j-1})+Scale\cdot ||{a}_{r1,*}-{a}_{h,*}|{|}_{2}.{\text{cos}}({\theta }_{j-1})j=dim.$$

In two-dimensional (2D) space, the spherical search can be formulated as:

5 $${a}_{i,j}^{g+1}={a}_{i,j}^{g}+Scale\cdot ||{a}_{pbest,*}-{\text{a}}_{i,*}|{|}_{2}.sin(\theta )+Scale\cdot |\left|{a}_{r1,*}-{\text{a}}_{h,*}\right|{|}_{2}.sin\left(\theta \right),$$

6 $${a}_{i,k}^{g+1}={a}_{i,k}^{g}+Scale\cdot ||{a}_{pbest,*}-{\text{a}}_{i,*}|{|}_{2}.cos({\theta })+Scale\cdot |\left|{a}_{r1,*}-{\text{a}}_{h,*}\right|{|}_{2}.cos\left(\theta \right),$$

where is a random number of uniform distribution between [0, 2$\mathrm\pi$].

${S}_{dim}$ numbers are selected randomly in the search space [1, 2, …, DIM], where DIM is the maximum number of SNPs, and ${S}_{dim}$ is the spherical search dimension (${S}_{dim}$=2 is the search in 2D space for 2-order SNP combinations or ${S}_{dim}$=3 is the search in 3D space for high-order SNP combinations). The search dimension can use 2D and 3D spherical search styles, with the two spherical search styles running alternately.

- Step 3: Updating operation

The fitness function of each new individual ${a}_{i}^{g+1}$  in a population is calculated and compared with the fitness function of the old individual $a_{i}^{g}$ . Excellent individuals are retained in a set *H*.

7 $${a}_{i}^{g+1}=\left\{\begin{array}{c}{a}_{i}^{g+1} \text{,} \, \, if \, {a}_{i}^{g+1}<{a}_{i}^{g}\\ {a}_{i}^{g} \, \, \end{array}\right.$$

- Step 4: Adaptive adjustment of spherical search scale

In spherical search operations, the spherical search factor is also known as the spherical search radius, which has a significant impact on the search of each individual. The traditional spherical search factor directly controls the size of the search radius based on the fitness function of the population. This method accelerates the convergence speed of the algorithm, but it also causes the algorithm to easily fall into local optima. Therefore, in this paper, we adopted the adaptive adjustment strategy for the spherical search factor as follows:

8 $$\Delta {f}_{i}^{g+1}=\left|f\left({a}_{i}^{g+1}\right)-f\left({a}_{i}^{g}\right)\right|$$

where $f\left({a}_{i}^{g+1}\right)$ is the fitness value of the new individual *i* in the population, $f\left({a}_{i}^{g}\right)$ is the fitness function value of the previous generation, and $\Delta {f}_{i}^{g+1}$ is the winning intensity of the individual.

9 $${w}_{i}^{g+1}=\frac{\Delta {f}_{i}^{g+1}}{{\sum }_{l=1}^{popsize}\Delta {f}_{l}^{g+1}}$$

where ${w}_{i}^{g+1}$ is the weight of the winning ability of the individual in the current generation of the population.

10 $$m{s}^{g+1}=\frac{ {\sum }_{n=1}^{popsize}{w}_{n}^{g+1}*(Scal{e}_{n}^{g}{)}^{2}}{{\sum }_{n=1}^{popsize}{w}_{n}^{g+1}*(Scal{e}_{n}^{g}{)}}$$

where ${\varvec{S}}{\varvec{c}}{\varvec{a}}{\varvec{l}}{{\varvec{e}}}_{{\varvec{n}}}^{{\varvec{g}}}$ is the value of the spherical search factor in generation $g$ and $m{s}^{g+1}$ is the weighted value of the spherical search factor of the population in generation $g+1$.

11 $$MS=\left[m{s}_{1},m{s}_{2},\dots ,m{s}_{r}\right]$$

where *MS* is a set of the weighted value of the spherical search factor for consecutive *r* generations.

12 $${t}_{i}=randc\left(m{s}_{r1},0.1\right)$$

A spherical search factor is selected randomly from the *MS* set and the Cauchy distribution, represented by $randc\left(m{s}_{r1},0.1\right),$ is used to generate a new search factor value ${t}_{i}$.

- Step 5: Population updating strategy

To enhance the diversity of the population, the excellent individuals who won in previous consecutive generations of the population are preserved and stored in a set of excellent individuals (*H*). In a spherical search operation, individual ${{\varvec{a}}}_{{\varvec{h}},{\varvec{j}}}$ are generated randomly from *H*, seen as Eqs. (5)– (6). ${H}^{\boldsymbol{^{\prime}}}$ is the new set of the current excellent individuals.

13 $${\varvec{H}}={\varvec{H}}\cup {{\varvec{H}}}^{\mathrm{^{\prime}}}$$

After each generation evolution, the next generation population size $Popsiz{e}_{g+1}$ is calculated as:

14 $$Popsiz{e}_{g+1}=round\left[\left(\frac{Popsiz{einit}_{min}}{FE{S}_{max}}\cdot FES+Popsiz{e}_{init}\right)\right]$$

$Popsiz{e}_{init}$ is set to the smallest possible value so that the reproduction operator can be applied; *FES* is the current fitness evaluation number and $FE{S}_{max}$ is the maximum fitness evaluation number.

### Linear mixed statistical epistasis model (LMSE)

In recent years, multi-objective algorithms have been used to solve EI problems. However, these algorithms do not converge efficiently for solving multi-objective optimization models. We combined two approximately normalized functions to convert a multi-objective optimization problem to a single-objective optimization problem for the evolutionary iterations of the algorithm, and calculated the lowest values of each $scoreFu{n}_{i}$ as:

15 $$LM={\sum }_{i=1}^{2}\frac{scoreFu{n}_{i}-Mi{n}_{i}}{Ma{x}_{i}-Mi{n}_{i}}$$

where $Ma{x}_{i}$ and $Mi{n}_{i}$ are estimates of the maximum and minimum values of $scoreFu{n}_{i}$ that are obtained during the initialization process.

**Fig. 9:**
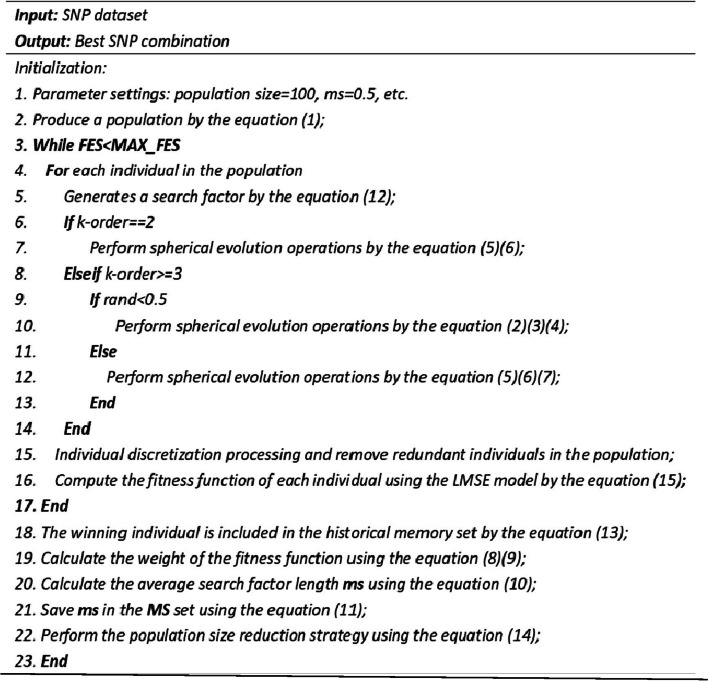
Pseudo code of the SEEI algorithm

This approach attempts to address the difficulty of convergence of multi-objective evolutionary algorithms to guarantee metric accuracy with high probability. Four statistical epistasis models were chosen to verify the performance of the proposed algorithm, namely K2-Score, LR, MI, and GINI. Pseudo code of the SEEI algorithm is provided in Fig. 9.

#### LR-Score [31]

LR is an indicator that reflects authenticity. It is a composite index that reflects both sensitivity and specificity and is calculated as:

16 $$LR=2\sum\nolimits_{i=1}^{I}\sum\nolimits_{j=1}^{J}{o}_{ij}{\text{ln}}\left(\frac{{o}_{ij}}{{e}_{ij}}\right)=2\sum\nolimits_{i=1}^{I}\sum\nolimits_{j=1}^{J}{n}_{ij}{\text{ln}}\left(\frac{{n}_{ij}}{{e}_{ij}}\right)$$

where ${o}_{ij}$ and ${e}_{ij}$ are the observed and expected genotype numbers, respectively, when SNPs are combined for genotype *j*, and phenotype is the disease state *i.* Low LR-Score values indicate strong association between a SNP combination and a phenotype.

#### K2-Score [31]

K2-Score, based on Bayesian networks, is calculated as:

17 $$K2-Score=\prod\nolimits_{i=1}^{I}\frac{\left(J-1\right)!}{{(N}_{i}+J-1)!}\prod\nolimits_{j=1}^{J}{N}_{ij}!,$$

where *I* is the number of all genotype combinations of a SNP and *J* is the number of all sample state sets. GWAS data usually contain only disease and control samples, so *J* is usually 2. ${N}_{i}$ is the number of SNP combinations of the ${i}_{th}$ genotype, and ${N}_{ij}$ is the number SNP combinations in the ${j}_{th}$ state. Low K2-Score values indicate high correlation of a SNP combination with a disease state.

#### Mutual information [32]

Mutual information (MI) is one of the most commonly used measures for feature selection. We used MI to measure the correlation between SNP combinations and phenotypes as:

18 $$MI\left(A;Y\right)=H\left(A\right)+H\left(A\right)-H\left(A,Y\right)$$

where *A* is the SNP combination, *Y* is the phenotype, *H(A)* is the entropy of *A*, *H(Y)* is the entropy of *Y*, and *H(A,Y)* is the joint entropy of *A* and *Y*. High MI scores indicate strong correlation between SNP combinations and phenotypes.

#### Gini index (GINI) [32]

The Gini index can be used to measure the degree of impurities and inequality in a dataset, as well as to analyze how dispersed the data are. The Gini Index is defined as:

19 $$GINI=\sum\nolimits_{i=1}^{I}{P}_{i}\left(1-\sum\nolimits_{j-1}^{J}{P}_{i,j}^{2}\right),$$

where ${P}_{i}$ is the probability of the ${i}_{th}$ genotype combination in the sample set, and ${P}_{i,j}$ is the estimated probability of the sample where the *i*-th genotype combination is related to phenotype *y*. $1-\sum_{j-1}^{J}{P}_{i,j}^{2}$ is the estimated probability that the genotype combination will be misclassified as phenotype *Y*. A low Gini coefficient indicates a high degree of equality and a strong association between a SNP combination and a phenotype.

**Fig. 10:**
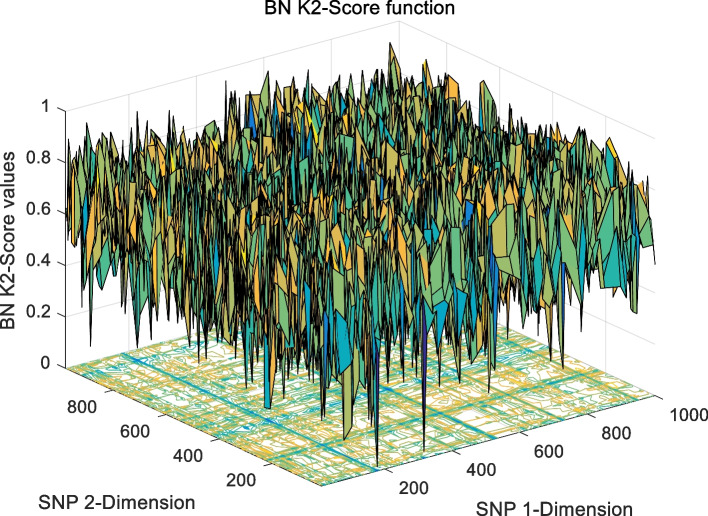
Single epistasis model BN K2 function

**Fig. 11:**
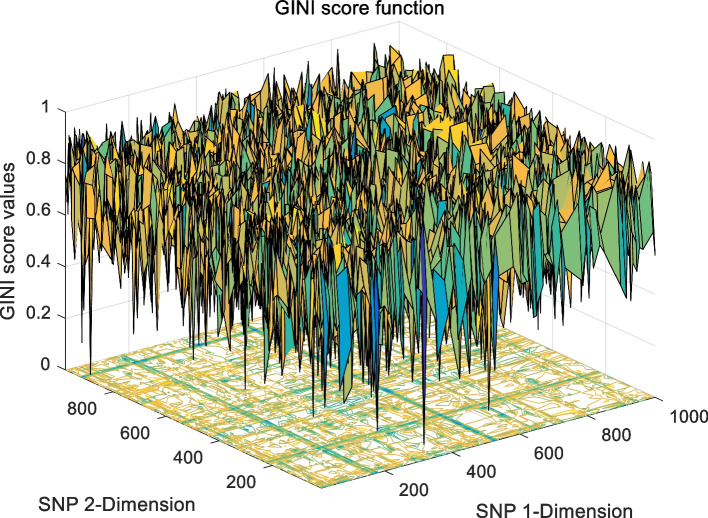
Single epistasis model GINI function

**Fig. 12:**
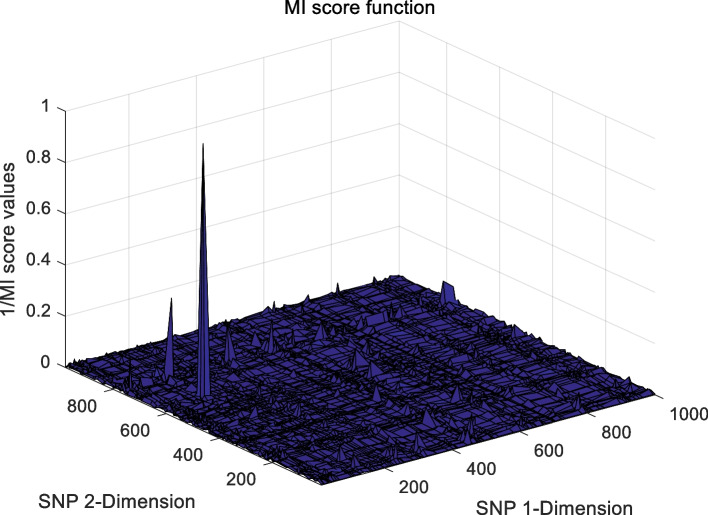
Single epistasis model MI function

**Fig. 13:**
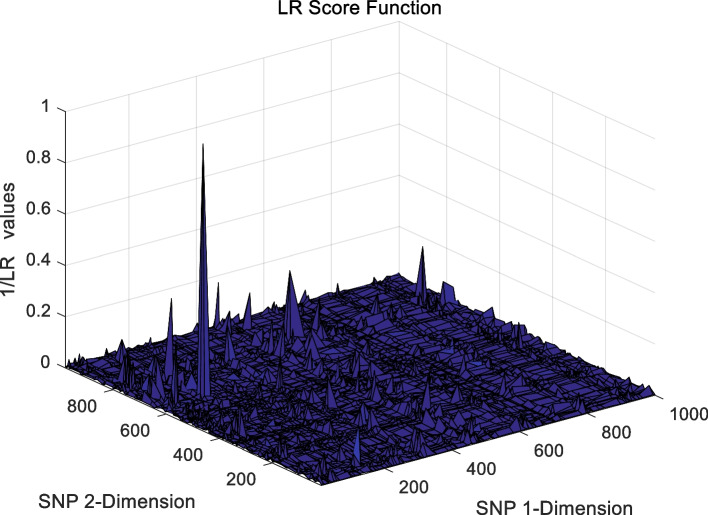
Single epistasis model LR function

**Fig. 14:**
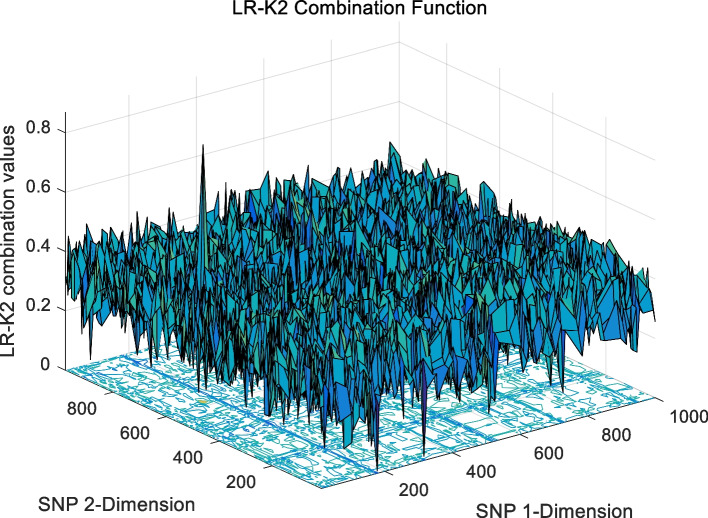
Linear mixed statistical epistasis model LR-K2 function

**Fig. 15:**
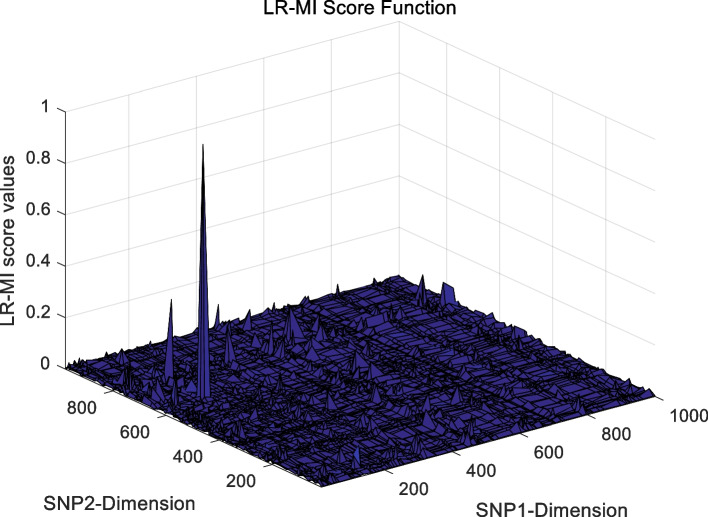
Linear mixed statistical epistasis model LR-MI function

**Fig. 16:**
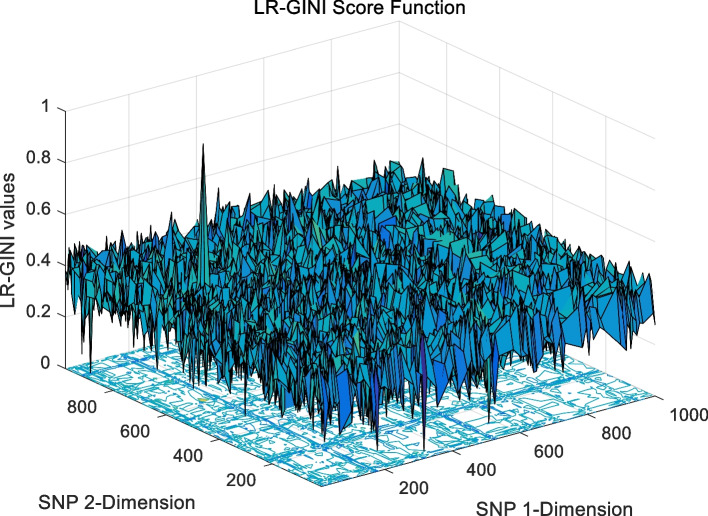
Linear mixed statistical epistasis model LR-GINI function

**Fig. 17:**
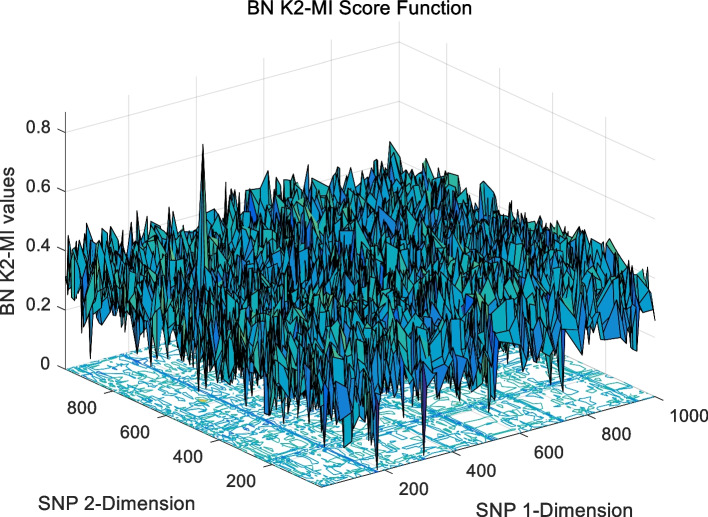
Linear mixed statistical epistasis model K2-MI function

**Fig. 18:**
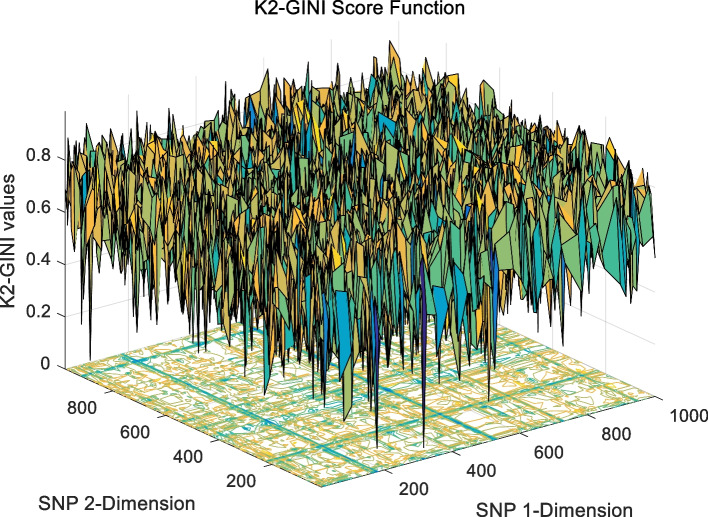
Linear mixed statistical epistasis model K2-GINI function

**Fig. 19:**
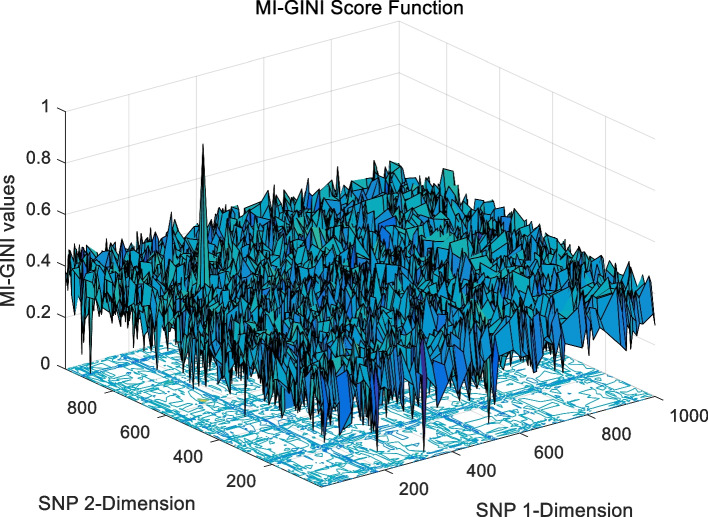
Linear mixed statistical epistasis model MI-GINI function

The landscape of 2-order SNP combinations that are constructed for the epistasis models by the first DME model (DME01 with 1000 SNPs and 400 samples) randomly produce 100 SNP combination pairs (2-order) in the 1000 SNPs. The search space is limited in a set as [1,2,…,1000] for each dimension. The values of the epistasis optimization model is as the fitness value, which is used to evaluate the association between SNP combinations and disease status. The fitness values of the four single epistasis models (LR, K2, MI, GINI) are shown in Figs. 10, 11, 12 and 13, and the fitness values of the six linear mixed statistical epistasis models (LR-K2, LR-MI, LR-GINI, K2-MI, K2-GINI, MI-GINI) are shown in Figs. 14, 15, 16, 17, 18 and 19. Each epistasis model can be considered an objective function with multiple local optima. The landscape of the linear mixed statistical epistasis model is similar to the single epistasis model. Indeed, the linear mixed statistical epistasis model convert the two-objective optimization problem into a single optimization problem.

## Supplementary Information

Supplementary Material 1.

## Data Availability

The Simulation datasets of the models were generated by GAMETES 2.0, that is available from https://surveillance.cancer.gov/genetic-simulation-resources/packages/gametes/.The simulation datasets can be produced by the GAMETES 2.0 according to the supplementary file. The Wellcome Trust Case Control Consortium (WTCCC) is a collaboration of many British research groups. The experimental protocols of the breast cancer (BC) dataset were approved by the Wellcome Trust Case Control Consortium. The consent was obtained from all subjects. The WTCCC data set in this study are available at https://www.wtccc.org.uk. The all datasets used during the current study available from the corresponding author on reasonable request.

## References

[1] Uitterlinden AG. An Introduction to Genome-Wide Association Studies: GWAS for Dummies. Semin Reprod Med. 2016;34(4):196–204. 27513020 10.1055/s-0036-1585406

[2] Dehghan A. Genome-Wide Association Studies. Methods Mol Biol. 2018;1793:37–49. 29876890 10.1007/978-1-4939-7868-7_4

[3] Tam V, Patel N, Turcotte M, Bossé Y, Paré G, Meyre D. Benefits and limitations of genome-wide association studies. Nat Rev Genet. 2019;20(8):467–84. 31068683 10.1038/s41576-019-0127-1

[4] Ding X, Wang J, Zelikovsky A, Guo X, Xie M, Pan Y. Searching High-Order SNP Combinations for Complex Diseases Based on Energy Distribution Difference. IEEE/ACM Trans Comput Biol Bioinform. 2015;12(3):695–704. 26357280 10.1109/TCBB.2014.2363459

[5] STOJANOVSKI TD. Performance of exhaustive search with parallel agents. Turk J Elect Eng Comp Sci. 2014;22:1382–94.

[6] Ritchie MD, Hahn LW, Roodi N, Bailey LR, Dupont WD, Parl FF, Moore JH. Multifactor-dimensionality reduction reveals high-order interactions among estrogen-metabolism genes in sporadic breast cancer. Am J Hum Genet. 2001;69(1):138–47. 11404819 10.1086/321276 PMC1226028

[7] Velez DR, White BC, Motsinger AA, Bush WS, Ritchie MD, Williams SM, Moore JH. A balanced accuracy function for epistasis modeling in imbalanced datasets using multifactor dimensionality reduction. Genet Epidemiol. 2007;31(4):306–15. 17323372 10.1002/gepi.20211

[8] Yang CH, Chuang LY, Lin YD. Multiobjective multifactor dimensionality reduction to detect SNP-SNP interactions. Bioinformatics. 2018;34(13):2228–36. 29471406 10.1093/bioinformatics/bty076

[9] Ritchie MD, Hahn LW, Moore JH. Power of multifactor dimensionality reduction for detecting gene-gene in teractions in the presence of genotyping error, missing data, phenocopy, and genetic heterogeneity. Genet Epidemiol. 2003;24(2):150–7. 12548676 10.1002/gepi.10218

[10] Yang CH, Lin YD, Chuang LY, Chen JB, Chang HW. MDR-ER: balancing functions for adjusting the ratio in risk classes and classification errors for imbalanced cases and controls using multifactor-dimensionality reduction. PLoS One. 2013;8(11):e79387. 24236125 10.1371/journal.pone.0079387 PMC3827354

[11] Lee S, Kwon MS, Oh JM, Park T. Gene-gene interaction analysis for the survival phenotype based on the Cox model. Bioinformatics. 2012;28(18):i582–8. 22962485 10.1093/bioinformatics/bts415 PMC3436842

[12] Yang CH, Hou MF, Chuang LY, Yang CS, Lin YD. Dimensionality reduction approach for many-objective epistasis analysis. Brief Bioinform. 2023;24(1):1–13. 10.1093/bib/bbac512 36458451

[13] Zhang Y, Liu JS. Bayesian inference of epistatic interactions in case-control studies. Nat Genet. 2007;39(9):1167–73. 17721534 10.1038/ng2110

[14] Shang J, Zhang J, Sun Y, Zhang Y. EpiMiner: A three-stage co-information based method for detecting and visualizing epistatic interactions. Digital Signal Processing. 2014;24:1–13.

[15] Flerova N, Marinescu R, Dechter R. Weighted heuristic anytime search: new schemes for optimization over graphical models. Ann Math Artif Intell. 2017;79(1–3):77–128.

[16] Tuo S. FDHE-IW: A Fast Approach for Detecting High-Order Epistasis in Genome-Wide Case-Control Studies. Genes (Basel). 2018;9(9):435. 30158504 10.3390/genes9090435 PMC6162554

[17] Wei C, Lu Q. GWGGI: software for genome-wide gene-gene interaction analysis. BMC Genet. 2014;15:101. 25318532 10.1186/s12863-014-0101-z PMC4201693

[18] Guy RT, Santago P, Langefeld CD. Bootstrap aggregating of alternating decision trees to detect sets of SNPs that associate with disease. Genet Epidemiol. 2012;36(2):99–106. 22851473 10.1002/gepi.21608 PMC3769952

[19] Wan X, Yang C, Yang Q, Xue H, Tang NL, Yu W. Predictive rule inference for epistatic interaction detection in genome-wide association studies. Bioinformatics. 2010;26(1):30–7. 19880365 10.1093/bioinformatics/btp622

[20] Wang X, Cao X, Feng Y, Guo M, Yu G, Wang J. ELSSI: parallel SNP-SNP interactions detection by ensemble multi-type detectors. Brief Bioinform. 2022;23(4):1–13. 10.1093/bib/bbac213 35696639

[21] Shang J, Cai X, Zhang T, Sun Y, Zhang Y, Liu J, Guan B. EpiReSIM: A resampling method of epistatic model without marginal effects using under-determined system of equations. Genes. 2022;13:2286. 36553553 10.3390/genes13122286 PMC9777644

[22] Sun Y, Gu Y, Ren Q, Li Y, Shang J, Liu J-X, Guan B. MDSN: A module detection method for identifying high-order epistatic interactions. Genes. 2022;13:2403. 36553670 10.3390/genes13122403 PMC9778340

[23] Wang J, Zhang H, Ren W, Guo M, Guoxian Yu. EpiMC: detecting epistatic interactions using multiple clusterings. IEEE/ACM Trans Comput Biol Bioinform. 2022;19(1):243–54. 33989157 10.1109/TCBB.2021.3080462

[24] David BB, Jan B, Markus H, Tim K, Markus L. A framework for modeling epistatic interaction. Bioinformatics. 2021;37(12):1708–16. 33252645 10.1093/bioinformatics/btaa990

[25] Uppu S, Krishna A. A deep hybrid model to detect multi-locus interacting SNPs in the presence of noise. Int J Med Inform. 2018;119:134–51. 30342681 10.1016/j.ijmedinf.2018.09.003

[26] Uppu S, Krishna A, Gopalan RP. A review on methods for detecting SNP interactions in high-dimensional genomic data. IEEE/ACM Trans Comput Biol Bioinform. 2018;15(2):599–612. 28060710 10.1109/TCBB.2016.2635125

[27] Wang X, Zhang H, Wang J, Yu G, Cui L, Guo M. EpiHNet: Detecting epistasis by heterogeneous molecule network. Methods. 2022;198:65–75. 34555529 10.1016/j.ymeth.2021.09.007

[28] Aflakparast M, Salimi H, Gerami A, Dubé MP, Visweswaran S, Masoudi-Nejad A. Cuckoo search epistasis: a new method for exploring significant genetic interactions. Heredity (Edinb). 2014;112(6):666–74. 24549111 10.1038/hdy.2014.4 PMC4023449

[29] Jing PJ, Shen HB. MACOED: a multi-objective ant colony optimization algorithm for SNP epistasis detection in genome-wide association studies. Bioinformatics. 2015;31(5):634–41. 25338719 10.1093/bioinformatics/btu702

[30] Sun Y, Shang J, Liu JX, Li S, Zheng CH. epiACO - a method for identifying epistasis based on ant Colony optimization algorithm. BioData Min. 2017;10:23. 28694848 10.1186/s13040-017-0143-7 PMC5500974

[31] Tuo S, Liu H, Chen H. Multipopulation harmony search algorithm for the detection of high-order SNP interactions. Bioinformatics. 2020;36(16):4389–98. 32227192 10.1093/bioinformatics/btaa215

[32] Sun Y, Wang X, Shang J, Liu JX, Zheng CH, Lei X. Introducing Heuristic Information Into Ant Colony Optimization Algorithm for Identifying Epistasis. IEEE/ACM Trans Comput Biol Bioinform. 2020;17(4):1253–61. 30403637 10.1109/TCBB.2018.2879673

[33] Tuo S, Zhang J, Yuan X, He Z, Liu Y, Liu Z. Niche harmony search algorithm for detecting complex disease associated high-order SNP combinations. Sci Rep. 2017;7(1):11529. 28912584 10.1038/s41598-017-11064-9 PMC5599559

[34] Li X, Zhang S, Wong KC. Nature-Inspired Multiobjective Epistasis Elucidation from Genome-Wide Association Studies. IEEE/ACM Trans Comput Biol Bioinform. 2020;17(1):226–37. 29994485 10.1109/TCBB.2018.2849759

[35] Wang H, Wu X. IPP: An Intelligent Privacy-Preserving Scheme for Detecting Interactions in Genome Association Studies. IEEE/ACM Trans Comput Biol Bioinform. 2023;20(1):455–64. 35239492 10.1109/TCBB.2022.3155774

[36] Peng YZ, Lin Y, Huang Y, Li Y, Luo G, Liao J. GEP-EpiSeeker: a gene expression programming-based method for epistatic interaction detection in genome-wide association studies. BMC Genomics. 2021;22(Suppl 1):910. 34930147 10.1186/s12864-021-08207-8 PMC8686218

[37] Gu Y, Sun Y, Shang J, Li F, Guan B, Liu JX. Multi-Objective Artificial Bee Colony Algorithm Based on Scale-Free Network for Epistasis Detection. Genes (Basel). 2022;13(5):871. 35627256 10.3390/genes13050871 PMC9140669

[38] Chen Y, Xu F, Pian C, Xu M, Kong L, Fang J, Li Z, Zhang L. E, EpiMOGA: An Epistasis Detection Method Based on a Multi-Objective Genetic Algorithm. Genes. 2021;12:191. 33525573 10.3390/genes12020191 PMC7911965

[39] M. G-CJ, Álvaro R-L, Sergio S-J, et al. Multiobjective evolutionary computation for high-order genetic interactions. Appl Soft Comput J. 2022;128:1–13.

[40] Tang D. Spherical evolution for solving continuous optimization problems. Appl Soft Comput J. 2019;81:105499.

[41] Yang C, He Z, Wan X, Yang Q, Xue H, Yu W. SNPHarvester: a filtering-based approach for detecting epistatic interactions in genome-wide association studies. Bioinformatics. 2009;25(4):504–11. 19098029 10.1093/bioinformatics/btn652

[42] Hoey J .The Two-Way Likelihood Ratio (G) Test and comparison to two-way chi squared test.Statistics. 2012. 10.48550/arXiv.1206.4881.

[43] Yang CH, Chuang LY, Lin YD. CMDR based differential evolution identifies the epistatic interaction in genome-wide association studies. Bioinformatics. 2017;33(15):2354–62. 28379338 10.1093/bioinformatics/btx163

[44] Urbanowicz RJ, Kiralis J, Sinnott-Armstrong NA, Heberling T, Fisher JM, Moore JH. GAMETES: a fast, direct algorithm for generating pure, strict, epistatic models with random architectures. BioData Min. 2012;5(1):16. 23025260 10.1186/1756-0381-5-16 PMC3605108

[45] Namkung J, Kim K, Yi S, Chung W, Kwon MS, Park T. New evaluation measures for multifactor dimensionality reduction classifiers in gene-gene interaction analysis. Bioinformatics. 2009;25(3):338–45. 19164302 10.1093/bioinformatics/btn629

[46] Ponte-Fernandez C, Gonzalez-Dominguez J, Carvajal-Rodriguez A, Martin MJ. Evaluation of existing methods for high-order epistasis detection. IEEE/ACM Trans Comput Biol Bioinform. 2022;19(2):912–26. 33055017 10.1109/TCBB.2020.3030312

[47] Derrac J, García S, Molina D, Herrera F. A practical tutorial on the use of nonparametric statistical tests as a methodology for comparing evolutionary and swarm intelligence algorithms. Swarm Evol Comput. 2011;1(1):3–18.

[48] Burton PR, Clayton DG, Cardon LR, Craddock N, Deloukas P, Duncanson A, Kwiatkowski DP, McCarthy MI, Ouwehand WH, Samani NJ, et al. Association scan of 14,500 nonsynonymous SNPs in four diseases identifies autoimmunity variants. Nat Genet. 2007;39(11):1329–37. 17952073 10.1038/ng.2007.17 PMC2680141

[49] Colletti JA 2nd, LelandWavrin KM, Kurz SG, Hickman MP, Seiler NL, Samanas NB, Eckert QA, Dennison KL, Ding L, Schaffer BS, et al. Validation of six genetic determinants of susceptibility to estrogen-induced mammary cancer in the rat and assessment of their relevance to breast cancer risk in humans. G3 (Bethesda). 2014;4(8):1385–94. 24875630 10.1534/g3.114.011163 PMC4132170

[50] Niu M, He Y, Xu J, Ding L, He T, Yi Y, Fu M, Guo R, Li F, Chen H, et al. Noncanonical TGF-β signaling leads to FBXO3-mediated degradation of ΔNp63α promoting breast cancer metastasis and poor clinical prognosis. PLoS Biol. 2021;19(2):e3001113. 33626035 10.1371/journal.pbio.3001113 PMC7939357

[51] Jansson M, Billing O, Herdenberg C, Lundin C, Tolockiene E, Nazemroaya A, Sund M. Expression and Circulating Levels of Perlecan in Breast Cancer - Implications for Oestrogen Dependent Stromal Remodeling. J Mammary Gland Biol Neoplasia. 2020;25(1):69–77. 32124140 10.1007/s10911-020-09447-2

[52] Clay MR, Varma S, West RB. MAST2 and NOTCH1 translocations in breast carcinoma and associated pre-invasive lesions. Hum Pathol. 2013;44(12):2837–44. 24140425 10.1016/j.humpath.2013.08.001

[53] Smith NL, Halliday BE, Finley JL, Wennerberg AE. The spectrum of immunohistochemical reactivity of monoclonal antibody DS6 in nongynecologic neoplasms. Appl Immunohistochem Mol Morphol. 2002;10(2):152–8. 12051634 10.1097/00129039-200206000-00010

[54] Tanabe R, Fukunaga A. Success-history based parameter adaptation for differential evolution. IEEE Congr Evol Comput. 2013;2013:71–8.

