# Supplementary material for: SEEI: spherical evolution with feedback mechanism for identifying epistatic interactions
Source: BMC Genomics. 2024 May 13;25:462. doi: 10.1186/s12864-024-10373-4 (PMC11633023; doi:10.1186/s12864-024-10373-4)
Supplement: Supplementary file 1 — Supplementary Material 1. [file 12864_2024_10373_MOESM1_ESM.docx]

# Supplementary Files

**SEEI: Spherical Evolution with Feedback Mechanism for Identifying Epistatic Interactions**

De-yu Tang^1,3*^, Yi-jun Mao^1*^, Jie Zhao^2^, Jin Yang^3*^, Shi-yin Li^3^, Fu-xiang Ren^3^, Junxi Zheng^3*^

1. Department of Computer Scienc, School of Mathematics and Informatics, School of Software Engineering, South China Agricultural University，Guangzhou 510642, PR China
2. School of Management, Guangdong University of Technology, Guangzhou 510006, PR China
3. School of Medical Information and Engineering, Guangdong Pharmaceutical University，Guangzhou 510006，PR China

*Correspondence: [scutdy@126.com](mailto:scutdy@126.com); zhengjunxi@gdpu.edu.cn

# 1 Disease models with marginal effect (DME)

**Table S1.** Model with marginal effects.

| *MAF* = 0.1, Prevalence = 0.050, *h*^2^ = 0.031 | | | | | | |
| --- | --- | --- | --- | --- | --- | --- |
| Model 1 | | AA | | Aa | | aa |
| BB | | 0.060 | | 0.010 | | 0.010 |
| Bb | | 0.010 | | 0.208 | | 0.208 |
| bb | | 0.010 | | 0.208 | | 0.208 |
| *MAF* = 0.1, Prevalence = 0.050, *h*^2^ = 0.014 | | | | | | |
| Model 2 | | AA | | Aa | | aa |
| BB | | 0.061 | | 0.017 | | 0.017 |
| Bb | | 0.017 | | 0.136 | | 0.136 |
| bb | | 0.017 | | 0.136 | | 0.136 |
| *MAF* = 0.1, Prevalence = 0.050, *h*^2^ = 0.01 | | | | | | |
| Model 3 | | AA | | Aa | | aa |
| BB | | 0.060 | | 0.021 | | 0.021 |
| Bb | | 0.021 | | 0.116 | | 0.116 |
| bb | | 0.021 | | 0.116 | | 0.116 |
| *MAF* = 0.1, Prevalence = 0.046, *h*^2^ = 0.016 | | | | | | |
| Model 4 | AA | | Aa | | aa | |
| BB | 0.030 | | 0.090 | | 0.070 | |
| Bb | 0.080 | | 0.010 | | 0.040 | |
| bb | 0.090 | | 0.010 | | 0.000 | |
| *MAF* = 0.1, Prevalence = 0.026, *h*^2^ = 0.009 | | | | | | |
| Model 5 | AA | | Aa | | aa | |
| BB | 0.030 | | 0.010 | | 0.020 | |
| Bb | 0.010 | | 0.090 | | 0.050 | |
| bb | 0.020 | | 0.050 | | 0.070 | |
| *MAF* = 0.1, Prevalence = 0.017, *h*^2^ = 0.008 | | | | | | |
| Model 6 | AA | | Aa | | aa | |
| BB | 0.020 | | 0.007 | | 0.003 | |
| Bb | 0.005 | | 0.070 | | 0.080 | |
| bb | 0.020 | | 0.001 | | 0.090 | |

**2 Disease models without marginal effect (DNME)**

**Table S2.** Models 1 to 10 without marginal effects.

| *h*^2^ = 0.2, *MAF* = 0.2 | | | | *h*^2^ = 0.2, *MAF* = 0.4 | | | |
| --- | --- | --- | --- | --- | --- | --- | --- |
| Model 1 | AA | Aa | aa | Model 6 | AA | Aa | aa |
| BB | 0.428 | 0.757 | 0.812 | BB | 0.356 | 0.891 | 0.809 |
| Bb | 0.788 | 0.132 | 0.044 | Bb | 0.955 | 0.508 | 0.611 |
| bb | 0.559 | 0.548 | 0.373 | bb | 0.617 | 0.755 | 0.63 |
| *h*^2^ = 0.2, *MAF* = 0.2 | | | | *h*^2^ = 0.2, *MAF* = 0.4 | | | |
| Model 2 | AA | Aa | aa | Model 7 | AA | Aa | aa |
| BB | 0.507 | 0.842 | 0.605 | BB | 0.086 | 0.536 | 0.641 |
| Bb | 0.845 | 0.162 | 0.629 | Bb | 0.677 | 0.275 | 0.096 |
| bb | 0.581 | 0.678 | 0.729 | bb | 0.219 | 0.413 | 0.712 |
| *h*^2^ = 0.2, *MAF* = 0.2 | | | | *h*^2^ = 0.2, *MAF* = 0.4 | | | |
| Model 3 | AA | Aa | aa | Model 8 | AA | Aa | aa |
| BB | 0.577 | 0.247 | 0.428 | BB | 0.855 | 0.339 | 0.772 |
| Bb | 0.227 | 0.928 | 0.578 | Bb | 0.513 | 0.651 | 0.607 |
| bb | 0.586 | 0.262 | 0.158 | bb | 0.25 | 0.999 | 0.154 |
| *h*^2^ = 0.2, *MAF* = 0.2 | | | | *h*^2^ = 0.2, *MAF* = 0.4 | | | |
| Model 4 | AA | Aa | aa | Model 9 | AA | Aa | aa |
| BB | 0.340 | 0.637 | 0.654 | BB | 0.506 | 0.838 | 0.024 |
| Bb | 0.689 | 0.017 | 0.041 | Bb | 0.603 | 0.454 | 0.957 |
| bb | 0.242 | 0.866 | 0.403 | bb | 0.729 | 0.427 | 0.753 |
| *h*^2^ = 0.2, *MAF* = 0.2 | | | | *h*^2^ = 0.2, *MAF* = 0.4 | | | |
| Model 5 | AA | Aa | aa | Model 10 | AA | Aa | aa |
| BB | 0.387 | 0.726 | 0.734 | BB | 0.393 | 0.764 | 0.664 |
| Bb | 0.749 | 0.090 | 0.034 | Bb | 0.850 | 0.398 | 0.733 |
| bb | 0.551 | 0.401 | 0.724 | bb | 0.406 | 0.927 | 0.147 |

**Table S2.** Models 11 to 20 without marginal effects

| *h*^2^ = 0.1, *MAF* = 0.2 | | | | *h*^2^ = 0.1, *MAF* = 0.4 | | | |
| --- | --- | --- | --- | --- | --- | --- | --- |
| Model 11 | AA | Aa | aa | Model 16 | AA | Aa | aa |
| BB | 0.463 | 0.703 | 0.431 | BB | 0.137 | 0.484 | 0.187 |
| Bb | 0.653 | 0.277 | 0.806 | Bb | 0.482 | 0.166 | 0.365 |
| bb | 0.830 | 0.008 | 0.129 | bb | 0.193 | 0.361 | 0.430 |
| *h*^2^ = 0.1, *MAF* = 0.2 | | | | *h*^2^ = 0.1, *MAF* = 0.4 | | | |
| Model 12 | AA | Aa | aa | Model 17 | AA | Aa | aa |
| BB | 0.319 | 0.507 | 0.569 | BB | 0.469 | 0.198 | 0.754 |
| Bb | 0.553 | 0.105 | 0.045 | Bb | 0.337 | 0.502 | 0.141 |
| bb | 0.203 | 0.777 | 0.280 | bb | 0.339 | 0.453 | 0.285 |
| *h*^2^ = 0.1, *MAF* = 0.2 | | | | *h*^2^ = 0.1, *MAF* = 0.4 | | | |
| Model 13 | AA | Aa | aa | Model 18 | AA | Aa | aa |
| BB | 0.627 | 0.393 | 0.335 | BB | 0.478 | 0.311 | 0.864 |
| Bb | 0.396 | 0.779 | 0.953 | Bb | 0.387 | 0.579 | 0.263 |
| bb | 0.174 | 0.842 | 0.106 | bb | 0.634 | 0.436 | 0.138 |
| *h*^2^ = 0.1, *MAF* = 0.2 | | | | *h*^2^ = 0.1, *MAF* = 0.4 | | | |
| Model 14 | AA | Aa | aa | Model 19 | AA | Aa | aa |
| BB | 0.297 | 0.54 | 0.441 | BB | 0.068 | 0.299 | 0.017 |
| Bb | 0.541 | 0.072 | 0.278 | Bb | 0.289 | 0.044 | 0.285 |
| bb | 0.434 | 0.293 | 0.228 | bb | 0.048 | 0.262 | 0.174 |
| *h*^2^ = 0.1, *MAF* = 0.2 | | | | *h*^2^ = 0.1, *MAF* = 0.4 | | | |
| Model 15 | AA | Aa | aa | Model 20 | AA | Aa | aa |
| BB | 0.332 | 0.562 | 0.573 | BB | 0.539 | 0.120 | 0.258 |
| Bb | 0.583 | 0.112 | 0.147 | Bb | 0.165 | 0.378 | 0.325 |
| bb | 0.399 | 0.496 | 0.033 | bb | 0.123 | 0.426 | 0.276 |

**Table S2.** Models 21 to 30 without marginal effects

| *h*^2^ = 0.05, *MAF* = 0.2 | | | | *h*^2^ = 0.05, *MAF* = 0.4 | | | |
| --- | --- | --- | --- | --- | --- | --- | --- |
| Model 21 | AA | Aa | aa | Model 26 | AA | Aa | aa |
| BB | 0.492 | 0.664 | 0.481 | BB | 0.002 | 0.155 | 0.214 |
| Bb | 0.642 | 0.330 | 0.746 | Bb | 0.199 | 0.071 | 0.022 |
| bb | 0.656 | 0.396 | 0.000 | bb | 0.081 | 0.122 | 0.135 |
| *h*^2^ = 0.05, *MAF* = 0.2 | | | | *h*^2^ = 0.05, *MAF* = 0.4 | | | |
| Model 22 | AA | Aa | aa | Model 27 | AA | Aa | aa |
| BB | 0.499 | 0.639 | 0.765 | BB | 0.188 | 0.020 | 0.171 |
| Bb | 0.666 | 0.389 | 0.083 | Bb | 0.032 | 0.174 | 0.059 |
| bb | 0.543 | 0.527 | 0.953 | bb | 0.134 | 0.087 | 0.092 |
| *h*^2^ = 0.05, *MAF* = 0.2 | | | | *h*^2^ = 0.05, *MAF* = 0.4 | | | |
| Model 23 | AA | Aa | aa | Model 28 | AA | Aa | aa |
| BB | 0.212 | 0.350 | 0.116 | BB | 0.005 | 0.179 | 0.251 |
| Bb | 0.336 | 0.054 | 0.495 | Bb | 0.211 | 0.100 | 0.026 |
| bb | 0.227 | 0.273 | 0.495 | bb | 0.156 | 0.098 | 0.156 |
| *h*^2^ = 0.05, *MAF* = 0.2 | | | | *h*^2^ = 0.05, *MAF* = 0.4 | | | |
| Model 24 | AA | Aa | aa | Model 29 | AA | Aa | aa |
| BB | 0.805 | 0.683 | 0.638 | BB | 0.174 | 0.321 | 0.154 |
| Bb | 0.657 | 0.936 | 0.989 | Bb | 0.223 | 0.254 | 0.245 |
| bb | 0.850 | 0.564 | 0.866 | bb | 0.448 | 0.025 | 0.424 |
| *h*^2^ = 0.05, *MAF* = 0.2 | | | | *h*^2^ = 0.05, *MAF* = 0.4 | | | |
| Model 25 | AA | Aa | aa | Model 30 | AA | Aa | aa |
| BB | 0.638 | 0.488 | 0.383 | BB | 0.098 | 0.219 | 0.302 |
| Bb | 0.464 | 0.765 | 0.957 | Bb | 0.302 | 0.126 | 0.121 |
| bb | 0.580 | 0.562 | 0.719 | bb | 0.053 | 0.308 | 0.136 |

**3 High-Order Disease Model**

**Table S3** High-Order Disease Model (Additive relationship)

| Oeder | MAF | P(D) | h² |
| --- | --- | --- | --- |
| 3 | 0.10 | 0.000012 | 0.10 |
| 3 | 0.10 | 0.000004 | 0.25 |
| 3 | 0.10 | 0.000002 | 0.50 |
| 3 | 0.10 | 0.000001 | 0.80 |
| 3 | 0.25 | 0.001153 | 0.25 |
| 3 | 0.40 | 0.022186 | 0.25 |
| 4 | 0.25 | 0.000068 | 0.25 |
| 4 | 0.40 | 0.003383 | 0.25 |
| 4 | 0.40 | 0.001374 | 0.50 |
| 4 | 0.40 | 0.000822 | 0.80 |
